# Methionyl-tRNA synthetase synthetic and proofreading activities are determinants of antibiotic persistence

**DOI:** 10.3389/fmicb.2024.1384552

**Published:** 2024-03-27

**Authors:** Whitney N. Wood, Miguel Angel Rubio, Lorenzo Eugenio Leiva, Gregory J. Phillips, Michael Ibba

**Affiliations:** ^1^Department of Microbiology, The Ohio State University, Columbus, OH, United States; ^2^Schmid College of Science and Technology, Chapman University, Orange, CA, United States; ^3^Department of Veterinary Microbiology, Iowa State University, Ames, IA, United States

**Keywords:** translation, tRNA, antibiotic resistance, persistence, aminoacyl-tRNA synthetase, methionine

## Abstract

Bacterial antibiotic persistence is a phenomenon where bacteria are exposed to an antibiotic and the majority of the population dies while a small subset enters a low metabolic, persistent, state and are able to survive. Once the antibiotic is removed the persistent population can resuscitate and continue growing. Several different molecular mechanisms and pathways have been implicated in this phenomenon. A common mechanism that may underly bacterial antibiotic persistence is perturbations in protein synthesis. To investigate this mechanism, we characterized four distinct *metG* mutants for their ability to increase antibiotic persistence. Two *metG* mutants encode changes near the catalytic site of MetRS and the other two mutants changes near the anticodon binding domain. Mutations in *metG* are of particular interest because MetRS is responsible for aminoacylation both initiator tRNA*^Met^* and elongator tRNA*^Met^* indicating that these mutants could impact translation initiation and/or translation elongation. We observed that all the *metG* mutants increased the level of antibiotic persistence as did reduced transcription levels of wild type *metG*. Although, the MetRS variants did not have an impact on MetRS activity itself, they did reduce translation rates. It was also observed that the MetRS variants affected the proofreading mechanism for homocysteine and that these mutants’ growth is hypersensitive to homocysteine. Taken together with previous findings, our data indicate that both reductions in cellular Met-tRNA*^Met^* synthetic capacity and reduced proofreading of homocysteine by MetRS variants are positive determinants for bacterial antibiotic persistence.

## 1 Introduction

Bacterial antibiotic persister cells are characterized by their ability to survive transient exposure to antibiotics without concomitant acquisition of genetic resistance (Lewis, 2007; Cotten and Davis, 2023). When exposed to antibiotics, most of the bacterial cells in a population are killed but a small proportion enter a non-growing, low metabolic state that can survive the antibiotic treatment. Once the antibiotic is removed, the persister cells can resuscitate and resume growth, eventually leading to repopulation of the bacterial culture (Lewis, 2007; Fisher et al., 2017; Balaban et al., 2019). Bacterial antibiotic persister cells are an important health concern as they are thought to be the cause of reoccurring bacterial infections and may also contribute to the emergence of stable antibiotic resistance (Balaban et al., 2013; Levin-Reisman et al., 2017, 2019; Cotten and Davis, 2023). Persistence differs from both resistance and tolerance in the mechanism by which it promotes survival in the presence of potentially toxic antibiotics. Bacterial antibiotic resistance is defined by an accompanying increase in a cell’s minimum inhibitory concentration (MIC) for a given antibiotic and is also inheritable. Consequently, a bacterial culture can continue to grow and divide in higher concentrations of an antibiotic because of the change in MIC. Bacterial antibiotic persistence is defined by a bimodal killing distribution. When an antibiotic is added to a bacterial population the entire population will initially be killed off at the same rate, while cells that are in a low metabolic state will be killed at a slower rate. Bacterial antibiotic tolerance is similar to antibiotic persistence, except that the entire population is killed at a slow rate while maintaining an unchanged MIC (Brauner et al., 2016; Balaban et al., 2019; Levin-Reisman et al., 2019). While a general model remains elusive for bacterial antibiotic persistence, several different mechanisms underlying this phenomenon have been described (Balaban et al., 2013; Radzikowski et al., 2017; Cotten and Davis, 2023).

Perturbed protein synthesis has recently gained attention as a potential common mechanism underlying several reports of bacterial antibiotic persistence (Kwan et al., 2013; van den Bergh et al., 2022). For example, mutations in several different aminoacyl-tRNA synthetases (aaRSs) in multiple bacterial species have been shown to increase the rates of antibiotic persistence (Girgis et al., 2012; Germain et al., 2013; Kaspy et al., 2013; Levin-Reisman et al., 2017; Silva-Valenzuela et al., 2017; Garoff et al., 2018; Yi et al., 2018; Hatch and Ouellette, 2020; Huang et al., 2020; Khare and Tavazoie, 2020; Wood et al., 2021). The aaRSs are responsible for correctly esterifying amino acids to their cognate tRNAs. This occurs in a two-step mechanism; first the amino acid is adenylated using ATP, second the adenylated amino acid is transferred to the cognate tRNA producing the aminoacyl-tRNA. The aminoacylated-tRNA then forms a ternary complex with EF-Tu and GTP which serves as a substrate for ribosomal protein synthesis (Ibba and Soll, 2000; Giege and Springer, 2016). Several aaRSs help to maintain the fidelity of protein synthesis via intrinsic proofreading mechanisms that check for and correct errors during cognate amino acid selection (Bullwinkle and Ibba, 2016; Mohler et al., 2018). AaRSs can utilize two different proofreading mechanisms. The first is pre-transfer editing, in which a misactivated non-cognate amino acid forms an aminoacyl adenylate which is subsequently proofread and hydrolyzed before it can be used to aminoacylate a tRNA. The second is post-transfer editing, in which a non-cognate aminoacyl-tRNA is proofread for an incorrect aminoacyl moiety prior to hydrolysis of the ester linkage and deacylation of the tRNA (Ibba and Soll, 2000; Giege and Springer, 2016; Pang et al., 2021). While recent studies have shown a direct role for the synthetic activities of aaRSs in persistence, the role of proofreading has remained less clear, in part due to its connection to the stringent response, itself a factor in determining persistence.

A previous study identified several different mutations in the *Escherichia coli metG* gene, which encodes methionyl-tRNA synthetase (MetRS), that increase the levels of bacterial antibiotic persistence (Slattery et al., 2013; Fridman et al., 2014). MetRS is unique as it plays a role in not only translation elongation, as do all aaRSs, but is also responsible for aminoacylation the translation initiator tRNA*^fMet^* (Ibba and Soll, 2000; Laursen et al., 2005). Furthermore, MetRS can pre-transfer edit homocysteine, a key intermediate in central metabolism (Jakubowski, 2003, 2017; Jakubowski and Glowacki, 2011; Ferla and Patrick, 2014). If intracellular levels of homocysteine become elevated, perturbation of MetRS’s proofreading activity could also alter levels of deacylated-tRNA*^Met^*. Elevated levels of deacylated tRNA have been shown to be determinants of bacterial antibiotic persistence (Kaspy et al., 2013; Wood et al., 2021), suggesting a direct link between central metabolism and persistence may be facilitated by MetRS.

In this study, we characterized four distinct *metG* mutants for their ability to increase antibiotic persistence. This included *metG*87, which has a point mutation near the catalytic site (S264F) and *metG*83, a double mutation also near the catalytic site (G263S/S264F) (Fridman et al., 2014). In addition, two mutants originally isolated as high persisters were investigated; *metG*630, originally isolated in *S. Typhimurium*, is a −4 frameshift mutation resulting in the change of 4 amino acids at the end of a truncated protein near the anticodon binding domain, and *metG*ΔETIT, a deletion mutation removing amino acids 569–572 also near the anticodon binding domain (Slattery et al., 2013). We have investigated how these *metG* mutations impact MetRS activity itself, as well as other aspects of protein synthesis such as translation initiation and elongation rates, polysome profiles, and proofreading of homocysteine both with and without initiator tRNA*^fMet^* and elongator tRNA*^Met^*. Taken together with previous findings, our data indicate that both reductions in cellular Met-tRNA*^Met^* synthetic capacity and reduced proofreading of homocysteine by MetRS variants are positive determinants for bacterial antibiotic persistence.

## 2 Materials and methods

### 2.1 Materials

*Escherichia coli* strains used in this study are shown in Supplementary Table 1. TransforMax™ EC100D™ pir + *E. coli* (LGC Biosearch Technologies) was used for construction of pRIPR plasmids and NEB5α (New England Biolabs) for all other plasmids. MFD*pir* (Ferrieres et al., 2010) was used as the donor strain for transfer of pRIPR plasmids by conjugation. All cells were made chemically competent using transformation and storage solution (Chung et al., 1989). Bacteria were grown using Miller Broth Base (Fisher Scientific) and supplemented with antibiotics ampicillin (Amp) (100 μg/mL), chloramphenicol (Cam) (30 μg/mL) and kanamycin (Kan) (30 μg/mL), (GoldBio). Other reagents included 5-bromo-4-chloro-3-indolyl-β-D-galactopyranoside (X-gal), Isopropyl-β-D-1-thiogalactopyranoside (IPTG), glucose (40% w/v), diaminopimelic acid (Fisher Scientific), and anhydrotetracycline-hydrochloride (aTc) (Sigma-Aldrich). Q5 High-Fidelity polymerase for PCR and Nt.BspQI nickase were obtained from New England Biolabs.

An assay using a penicillinase from *Bacillus cereus* (Sigma-Aldrich) was used to test recombinants for antibiotic persistence and conducted as described previously (Slattery et al., 2013). DNA purifications were performed using Monarch PCR and DNA Cleanup Kit and Monarch Plasmid Miniprep Kit (New England Biolabs). Genomic DNA extractions were generated with MasterPure Complete DNA and RNA Purification Kit (formerly Epicentre) with Sanger sequencing carried out at the Iowa State University DNA Facility. Primers and synthetic DNA fragments were ordered from Integrated DNA Technologies, Coralville, IA, USA.

### 2.2 Construction of pRIPR vectors

**FIGURE 1:**
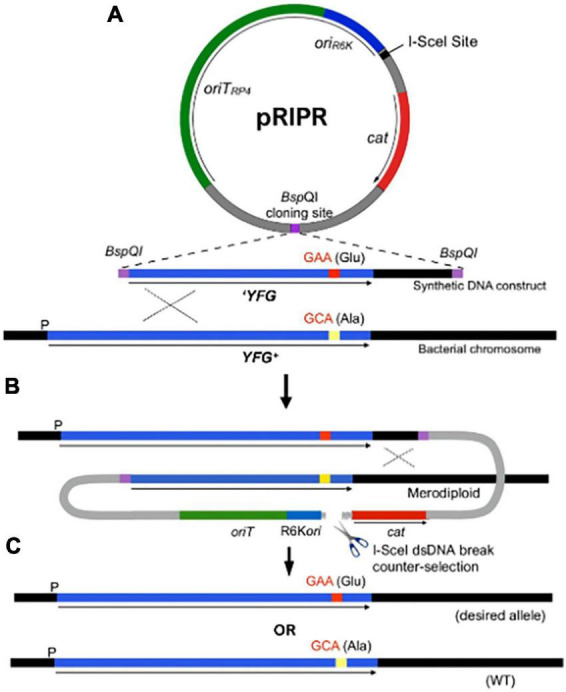
The R6K integrated-plasmid recombination (RIPR) system for genome editing by allelic exchange. **(A)** The pRIPR vector contains π dependent R6K origin of replication (*oriR6K*), origin of transfer *oriT*_*RP4*_ and the 18-bp I-SceI meganuclease recognition site. Restriction enzyme recognition sites (e.g., BspQI) facilitate insertion of DNA containing the desired sequence change (e.g., GAA, encoding glutamate [Glu]) flanked by homology arms (YFG). Plasmid transformants and recombinants are selected by chloramphenicol resistance (*cam*). **(B)** Following conjugation into a recipient cell, pRIPR integrates into the bacterial chromosome by a single-crossover event. Production of I-SceI from the helper plasmid pSceH (not shown) generates a double-stranded DNA break as a counterselection against plasmid integrates. **(C)** Resolution of the cointegrate by a second recombination event results in the loss of RIPR construct leaving behind the desired sequence change (GAA) or the wild type sequence (GCA).

The R6Kγ*ori* plasmid pVIK107 (Kalogeraki and Winans, 1997) was modified by first deleting the *lac* operon genes on the vector by inverse PCR using primers VIKdel.S and VIKdel.AS (Supplementary Table 2). Primers were synthesized with a 5′ phosphate when used for inverse PCR reactions. The 6.3-kbp linearized PCR product was gel purified, re-circularized by ligation and transformed into EC100D™ pir+. Next, a 1-kbp *cat* cassette was amplified from pKD3 (Datsenko and Wanner, 2000) using primers KD3-Sce-SacI.S and KD3-Sce-SacI.AS, which introduced an I-SceI meganuclease recognition site at the 5′ end, along with SacI restriction sites at the 5′ and 3′ ends of the PCR products. The purified cassette was then introduced into the modified pVIK107 by digestion with SacI and ligation. Inverse PCR was then performed directly upstream and downstream of the *neo* gene selectable marker with primer pair VIK-NheI.S and VIK-NheI.AS to introduce flanking NheI restriction sites. The linear PCR product was gel purified, digested with NheI and re-circularized by ligation, resulting in the replacement of the *neo* gene with a single NheI site. We then generated a *kan* cassette using pKD4 (Baba et al., 2006) as a template using primers BspQI-KD4.S and BspQI-KD4.AS to introduce BspQI and StuI restriction sites. The PCR products were purified, digested with NheI and ligated into the modified pVIK107 vector to yield pRIPR (Figure 1).

To generate derivatives of pRIPR for genome editing, DNA fragments containing the desired mutation, including specific base pair changes or deletions flanked by homologous DNA, were created by synthetic gBlock gene fragments (Integrated DNA Technologies, Coralville, IA, USA) or by PCR amplification of *E. coli* genomic DNA. To facilitate cloning into pRIPR, we used ligation-independent cloning using Nt.BspQI nickase enzyme, as described previously (Oster and Phillips, 2011). For this, BspQI nickase recognition sites were introduced to the 5′ ends of gBlocks and PCR products using primers with 5′ extensions that included the enzyme sites (Supplementary Table 2). pRIPR was linearized with StuI to remove the Kan*^R^* cassette and cloning reactions were transformed into EC100D™ pir+ and colonies selected on LB agar plates containing Cam. Transformants were patched on LB agar + Kan to test for loss of Kan*^R^*. Plasmid DNA isolated from Kan*^S^* colonies and the presence of an insert of correct size were confirmed by NheI digestion.

### 2.3 Construction of helper plasmid pSceH

Since the RIPR technique does not require exogenous recombinases, the plasmid pSLTS (Kim et al., 2014) was digested with blunt-end restriction enzymes EcoRV and StuI and religated to remove the λ-Red recombineering genes (*gam*, *beta*, and *exo*) to yield pSceH.

### 2.4 Genome editing using the RIPR method

pRIPR constructs were generated to target either *lac* or *metG* on the *E. coli* chromosome for mutagenesis. pRIPR-derivative plasmids were transformed into MFD*pir* by selecting colonies on LB agar plates supplemented with Cam and 150 mM DAP. Transformants were used as donors in conjugation reactions with BW30270 recipients as described (Ferrieres et al., 2010).

Cam*^R^* recombinants were colony purified and transformed with the helper plasmid pSceH by selection on LB + Amp plates spread with 50 μL of anhydrotetracycline (100 μg/mL) and incubated overnight at 30°C. Single colonies were then purified by streaking onto LB and LB+Cam plates to screen for recombinants that had lost Cam*^R^*. Colonies were again restreaked at 37°C and tested to ensure loss of pSceH. Recombinants were tested for new phenotypes by culturing on LB plates spread with X-Gal and IPTG (Supplementary Figure 1) to identify the desired recombinants and *metG* mutants tested by penicillinase persister assays (Slattery et al., 2013). To further confirm the phenotypes of high persister mutants, the tolerance disk test (TD test) was performed (Gefen et al., 2017). All recombinants were also tested by PCR and Sanger sequencing to confirm the predicted genetic changes.

### 2.5 CRISPR interference strains

For CRISPRi experiments, single guide RNA (sgRNA) plasmids were constructed using a derivative of pgRNA-bacteria (Qi et al., 2013) modified to replace the ColE1 replication origin with the ColE1-compatible replicon from RSF1030 (Phillips et al., 2000). Twenty base pair sequences specifying guide RNAs targeting the aaRS genes for alanine (*alaS*) and methionine (*metG*) were introduced by inverse PCR (Supplementary Table 2) and religation. Plasmids were confirmed by DNA sequencing and then transformed into LC-E75 (Cui et al., 2018) for phenotypic characterization.

### 2.6 Growth curves and colony forming units (CFU)

Growth curves for wild-type *E. coli* and aminoacyl-tRNA synthetase CRISPRi knock down strains were performed by starting overnight cultures in Lysogeny broth (LB) medium with no antibiotic added. The next morning overnight cultures were diluted 1:100 into fresh LB medium and were aliquoted into a 96-well plate. Growth curves were started in a Tecan Spark microplate reader at 37°C, with the plate shaking, and optical density (OD) was measured at 600 nm every 15 min for 20 h. After 1 h of growth, anhydrous tetracycline (aTc) was added to the 96-well plate to final concentrations of 0, 0.25, 0.5, 1.0, 1.5, 2.0, 2.5, and 4.0 μg/mL. Growth curves for wild-type *E. coli* and *metG* variant strains were measured by growing cultures in LB medium with a 1:100 dilution from an overnight culture. The OD at 600 nm was measured every 30 min for 8 h. Colony forming units were measured against different concentrations of aTc to determine the optimal knock down efficiency of each individual guide RNA used for CRISPRi knock down. Briefly, overnight cultures were started the same as with the growth curves. Cultures were grown in LB medium with 1:100 dilution from an overnight culture. Bacterial cultures were grown at 37°C, shaking at 250 rpm. After 1 h of growth, aTc was added to the culture to a final concentration of 0, 0.5, 1.0, 1.5, 2.0, and 2.5 μg/mL. The cultures continued to grow for another 3 h with shaking. At this point the OD at 600 nm was measured and 1 mL of culture was taken out and serial diluted and plated on LB plates to count CFU/mL for each concentration of aTc used.

### 2.7 Minimum duration of killing (MDK) and endpoint persister assays

Minimum duration of killing (MDK) bacterial persistence assays were performed for wild-type and variant *metG E. coli* as previously described (Wood et al., 2021). Endpoint bacterial persistence assays were performed for the aminoacyl-tRNA synthetase CRISPRi knock down strains (wild-type, empty vector, alaS and metG) by starting an overnight culture in LB with no addition of antibiotic. The next morning cultures were started in fresh LB with a 1:100 dilution from the overnight culture. Cultures were grown at 37°C, shaking at 250 rpm, until the OD at 600 nm reached ∼0.1 (∼1 h). aTc was added to a final concentration of 2.0 μg/mL for wild-type and empty vector strains, and 0.5 μg/mL for the *alaS* and *metG* knock down strains (determined by growth curves and CFU/mL). The cultures continued to grow at 37°C with shaking for another 3 h. At this point 1 mL of culture was taken out and serial diluted and plated on LB plates to count colony forming units (CFU). HA filter disks were placed on LB plates supplemented with 100 μg/mL ampicillin and 100 mL of a 1:10 dilution was aliquoted onto the filter disk. These plates were incubated overnight at 37°C. The next day the filter disks were transferred to fresh LB plates and incubated overnight again at 37°C. Colonies were then counted to obtain CFU. To calculate persisters the CFU of survivors after ampicillin treatment was divided by the CFU of the culture with no ampicillin treatment.

### 2.8 ^35^S-methionine protein synthesis rate assay

Overnight cultures of the wild-type and *metG* varient strains were grown in M9 minimal media supplemented with 50 μM Methionine and back-diluted to an OD at 600 nm of 0.05 in the same media. Cultures were grown at 37°C until they reached an OD at 600 nm of 0.7, at which point 5 μL of 35S-Methionine was added (10.25 mCi/mL). From this point onward, aliquots of 50 μL were taken every 2 min, dispersed onto a filter pad soaked with 5% trichloroacetic acid (TCA) plus 5 μM methionine and washed 3 times in 5% TCA. Radioactivity was counted by a liquid scintillation counter (Beckman Coulter LS 6500 scintillation system).

### 2.9 Polysome profiles

Polysome profiles for wild-type and variant metG *E. coli* were performed as previously described (Qin and Fredrick, 2013). Briefly, cultures were grown overnight in M9 minimal media supplemented with 50 μM methionine and back diluted to an OD at 600 nm of 0.05 in the same media. Cultures were grown at 37°C to an OD at 600 nm of 0.7, cooled rapidly by mixing with crushed ice and collected by centrifugation. Cellular pellets were resuspended in 300 mL lysis buffer (10 mM Tris–HCl, pH 8.0, 1 mM MgCl2, 1 mg/mL Lysozyme) and flash frozen in liquid nitrogen. Samples were thawed, mixed with 10% sodium deoxycholate and flash frozen again. After thawing, samples were centrifuged at 10000 rpm, 4°C for 10 min and the supernatant was loaded on top of a 10–40% sucrose gradient.

The gradients were centrifuged at 35000 rpm, 4°C for 3 h in a SW41 swinging buckets rotor. After centrifugation, the gradient tubes were mounted onto a Brandel/ISCO syringe-pump system equipped with a UV detector and the contents were injected through the system at a rate of 1.5 mL/min. Absorbance at 254 nm was monitored during the process, yielding the polysome profile. Ribosomes corresponding to the 30S, 50S, 70S and polysome fractions were calculated as a percentage of the total area.

### 2.10 Determination of the initial observation time of GFP

The comparison of the translational initiation rate was based on the determination of the initial observation time of GFP. *metG* mutants and their parental strain were transformed with plasmid pS1, a low copy number encoding a *gfp* gene under an inducible arabinose promoter (Leiva et al., 2022). Briefly, saturated overnight cultures in LB media with ampicillin (100 g/mL) were washed and diluted to the same 0.1 OD_600_ in fresh M9 media (7.7 mM Na_2_HPO_4_, 22.0 mM KH_2_PO_4_, 8.6 mM NaCl, 18.7 mM NH_4_Cl, 2 mM MgSO_4_, 0.1 mM CaCl_2_, and 0.4% Glycerol) with ampicillin (M9 amp). In a 96-well optical-bottom plate, 100 μl of each strain were incubated for 30 min at 37°C, to then add 100 μl of M9 amp supplemented with arabinose (0.4% final concentration). Plates were further shaken at 37°C in a microplate reader (SPARK, TECAN) where OD_600_ and GFP fluorescence intensity (Ex. 480 ± 20 nm, Em. 525 ± 20 nm) were determined. The square root of the fluorescence data and time (Schleif plot) were used to obtain the initial observation time of GFP (T_*first*_ GFP) in each strain (Schleif et al., 1973).

### 2.11 tRNA plasmid construction

*Escherichia coli* tRNA*^Met^* and tRNA*^fMet^* encoding genes were individually cloned into a pUC18 plasmid. To generate the tRNA insert six overlapping synthetic DNA oligonucleotides that encode the T7 RNA promoter and the tRNA gene were heated to 90°C for 5 min and slowly cooled to room temperature. The annealed fragments were then ligated and cloned into pUC18 using the EcoRI and XbaI restriction digest sites. The first nucleotide in tRNA*^fMet^* was mutated from cytosine to guanosine for T7 RNA polymerase to start transcription (G1-tRNA*^fMet^*). Plasmid sequence was confirmed by DNA sequencing. A pUC18 plasmid with tRNA*^fMet^* containing its natural first cytosine was ordered from GenScript Biotech Corp (Piscataway, NJ, USA). A hammerhead ribozyme sequence was placed between the T7 promoter and the tRNA sequence to generate a natural tRNA*^fMet^* isoacceptor (C1-tRNA*^fMet^*) (Fechter et al., 1998).

### 2.12 *In vitro* tRNA transcription

*In vitro* transcription of tRNA*^Met^* and tRNA*^fMet^* were performed as described (Steiner et al., 2019). Briefly, plasmids containing *E. coli* tRNA*^Met^* and tRNA*^fMet^* were used as template for PCR amplification of T7 promoter and tRNA. The PCR-amplified product was purified and used for T7 runoff transcription. *In vitro* transcription was performed using the purified PCR product and T7 RNA polymerase suspended in a buffer comprised of 40 mM Tris–HCl, Ph 8.0, 2 mM spermidine, 22 mM MgCl_2_, 5 mM DTT, 50 μg/mL BSA, 4 mM ATP, 4 mM GTP, 4 mM CTP, 4 mM UTP, 20 mM 5′-GMP, pyrophosphatase and RNase inhibitor. Reactions were incubated overnight in a 42°C water bath. The tRNA products were purified using DEAE cellulose anion exchange resin and eluted with 1 M NaCl. The fractions that contained tRNA were monitored by absorbance at 260 nm and pooled together and ethanol precipitated overnight at −20C. The ethanol precipitated tRNA was centrifuged at 4,500 × *g* for 10 min at 4°C. The resulting pellet was dried and suspended in RNase free water. For *in vitro* transcription of tRNA*^fMet^* with its natural cytosine at the first position and containing the hammerhead ribozyme between the T7 promoter and the tRNA sequence a few modifications were made to the purification of the tRNA (Fechter et al., 1998). After *in vitro* transcription was complete, 8 mM MgCl_2_ was added to the reaction, which was then diluted fivefold with RNase free water and incubated at 60°C for 2 h. This allows for the hammerhead ribozyme to cleave before the first cytosine in tRNA*^fMet^*, releasing its natural isoacceptor (C1-tRNA*^fMet^*). This reaction was then ethanol precipitated. The resulting pellet was dried and suspended in RNase free water. The C1-tRNA*^fMet^* was resolved on an 8% acrylamide, 9 M urea gel in Tris-boric acid-EDTA (TBE) buffer at 300 volts for 30 min. Under ultraviolet light the different tRNA fragments will illuminate. The middle fragment was excised from the gel (top fragment is the full transcript, the middle fragment is C1-tRNA*^fMet^*, and the bottom fragment is the hammerhead ribozyme). The excised gel piece was cut up and added to a polyprep column containing 0.5 M ammonium acetate, 0.1% SDS, and 5 mM EDTA, pH 8.0, and incubated overnight at room temperature with agitation. The next day, the tRNA was eluted from the polyprep column and ethanol precipitated. The concentration of active tRNA was determined using aminoacylation kinetics as described below.

### 2.13 Steady-state aminoacylation kinetics and ATP/PPi exchange

His_6_-tagged wild-type and variant MetRS proteins were expressed from a pET28a plasmid in an *E. coli* BL21 (DE3) RIL strain. Protein expression and purification was performed essentially as described (Roy et al., 2005). The concentration of active MetRS was determined by active site titrations, as described (Ibba et al., 1994). To determine the steady-state amino acid activation kinetics for wild-type and variant MetRS ATP/PPi exchange assays were done as previously described (Roy et al., 2005). Briefly, reactions were performed at 37°C in 100 mM Na-HEPES, pH 7.2, 30 mM KCl, 10 mM MgCl_2_, 2 mM NaF, 2 mM ATP, 2 mM ^32^P-PPi (2 cpm/pmol) with various amounts of methionine (0–400 μM), and 25–100 nM enzyme. After 1–4 min, 25 μL of the reaction was quenched in a solution containing 1% activated charcoal, 5.6% HClO_4_, and 75 mM PPi. The ^32^P-ATP bound-charcoal was filtered through a 3 mm Whatman disc filter under vacuum and washed 3 times with 5 mL water. The filters were dried, and the radioactivity was counted by a liquid scintillation counter (Beckman Coulter LS 6500 scintillation system). Steady-state aminoacylation kinetics were performed as described (Francklyn et al., 2008). Briefly, reactions were performed in 100 mM Na-HEPES, pH 7.2, 20 mM KCl, 30 mM MgCl_2_, 0.1 mg/mL BSA, 0.5 mM DTT, 40 mM ^14^C-L-methionine, and 50 nM enzyme. Various concentrations of tRNA*^Met^* and tRNA*^fMet^* (0 20 μM) and ATP (0–10 mM) were used depending on the substrate being tested for steady-state kinetics. Reactions were incubated at 30°C and at different minute intervals an aliquot of reaction was quenched on a filter pad soaked with 5% trichloroacetic acid (TCA) plus 5 μM methionine. The filters were washed 3 times in 5% TCA for 5 min each, dried, and the radioactivity was quantified by liquid scintillation counting. Kinetics measurements were calculated using GraphPad Prism.

### 2.14 S-NO-homocysteine synthesis

S-NO-homocysteine was synthesized as previously described (Stamler et al., 1993; Jakubowski, 2000). Briefly, equimolar amounts of L-homocysteine and NaNO_2_ were dissolved in 0.1 M HCl and incubated at 25°C. The reaction was neutralized with 0.5 M Na_2_HPO_4_, pH 7.4, and is complete when the reaction turns red. A small aliquot of the S-NO-homocysteine reaction and L-homocysteine were run on a silica TLC in *n*-butanol:acetic acid: ethyl acetate:water (1:1:1:1). After the TLC is complete and let dry, the amino acids are stained with a ninhydrin spray. S-NO-homocysteine will migrate faster than L-homocysteine. S-NO-homocysteine can be stored at −80°C for up to 2 weeks.

### 2.15 ATP futile cycling assays

ATP consumption assays were performed to analyze pre-transfer editing of L-homocysteine and S-NO-homocysteine by wild-type and variant MetRS mutants as previously described (Steiner et al., 2019). Reactions contained 20 μM tRNA*^Met^* or C1-tRNA*^fMet^* (natural tRNA*^fMet^* isoacceptor), 10 mM methionine, L-homocysteine, or S-NO-homocysteine, 3 mM ^32^P-γ-ATP (∼5 CPM/pmole), 2 U/μL pyrophosphatase, and 1 μM wild-type or variant MetRS in a buffer containing 100 mM Na-Hepes, pH 7.2, 30 mM KCl, and 10 mM MgCl_2_. Reactions were performed at 37°C and aliquots were quenched in an equal volume of glacial acetic acid at 3, 6, 10, 15, 20, and 30 min time points. A PEI cellulose TLC plate was pre-run in water, let dry, spot 1 μL of the quenched timepoint, and run in 0.7 M K_2_HPO_4_, pH 9 until the buffer reaches ∼1 cm from the top of the TLC plate. Let the TLC dry and expose to a phosphor screen and imaged on a phosphor imager (Typhoon). Quantification of the percentage of inorganic phosphate was completed using ImageQuant and GraphPad Prism.

### 2.16 Growth rates with homocysteine

For all growth experiments, overnight cultures of the wild-type and variant *metG* strains were grown to saturation in M9 minimal medium supplemented with 50 μM methionine. Cultures were collected by centrifugation, washed with fresh M9 media and backdiluted to an OD at 600 nm of 0.05 in M9 media supplemented with either Homocysteine. Cultures were then incubated at 37°C for 18 h, at which point the OD at 600 nm was measured. Four biological replicates were measured for each condition.

## 3 Results

### 3.1 Development of a pRIPR allelic exchange system

To facilitate construction of the *metG* mutants characterized in this study, we developed a gene editing system that allowed for specific base pair changes to be made to the *E. coli* chromosome. While several methods have been reported for genome sequence modifications, including λ Red-mediated homologous recombination (Datsenko and Wanner, 2000; Yu et al., 2000) and CRISPR-Cas9 technologies (Backes and Phillips, 2021) none is ideally suited for our specific needs. We, therefore, sought to develop a genetic system with no limitations as to the sequence changes made, constraints on surrounding sequences or host genotype that could be applied to genes essential for viability at high efficiency with a minimum number of steps.

Our approach was to build on the well-established R6K-based suicide vector system (Phillips, 2004), in combination with a proven counterselection strategy afforded by expression of the I-SceI meganuclease to generate lethal chromosomal breaks (Posfai et al., 1999; Datsenko and Wanner, 2000; Wong and Mekalanos, 2000; Lee et al., 2009). One limitation of R6K-based system is the requirement for relatively long regions of homology that cannot easily be incorporated into PCR primers. However, by taking advantage of the current availability of synthetic DNA constructs that can be custom made at low cost, along with methods that facilitate construction of recombinant plasmids (Casini et al., 2014), Since suicide vector systems offer several advantages for genome editing, we developed the R6K integrated-plasmid recombination (RIPR) system (summarized in Figure 1) for scarless editing of both essential and non-essential genes and to specifically investigate molecular mechanisms of MetRS activity in the phenomena of antibiotic persistence. The efficiency of the RIPR system for gene editing was validated by conducting several genome edits of the *lac* operon of *E. coli* (see Supplementary Information Results).

### 3.2 Use of RIPR to construct *metG* mutants

We used the RIPR system to construct *E. coli* mutants expressing different variants of *metG* to assess their contributions to bacterial antibiotic persistence. Given that bacterial cells that have entered an antibiotic persistent state represent only a small subpopulation of a culture, genetic approaches to study the phenomenon have included isolation of high persister mutants. Interestingly, *metG* mutants have been isolated in multiple studies seeking high persisters, including in *E. coli* (Girgis et al., 2012; Fridman et al., 2014; Levin-Reisman et al., 2017), *Salmonella enterica* (Slattery et al., 2013), *Shigella flexneri* (Zhu et al., 2018), *Burkholderia thailandensis* (Yi et al., 2018), and *Vibrio cholerae* (Silva-Valenzuela et al., 2017). While the function of MetRS in protein synthesis has been well characterized (Dardel et al., 1984; Ibba and Soll, 2000), its function in persistence is not well understood (Girgis et al., 2012; Harms et al., 2016).

For our investigation, we used RIPR to engineer four *metG* alleles identified from multiple sources into wild type *E. coli* K-12 (Supplementary Table 1). The *metG* mutations include *metG*Δ*ETIT*, which originated from an *E. coli* mutant isolated in a screen for antibiotic tolerance that results in an internal deletion of amino acids 569–572 (Levin-Reisman et al., 2017) and *metG630*, a frameshift mutation near the 3’ end of the gene that is comparable to a *metG* variant isolated as a high persister mutant in *S. enterica* (Slattery et al., 2013). In addition, alleles *metG83* (Blumenthal, 1972) and *metG87* (Greene et al., 1973) were characterized. DNA sequence analyses revealed *metG83* corresponds to amino acid changes G263S and S264F and *metG87* with S264F. These mutants were originally isolated as methionine auxotrophs and have not previously been characterized for antibiotic persistence. To construct these mutants, we included the 3’ end of the *metG* gene as part of PCR products (*metG83* and *metG87*) and synthetic DNA constructs (*metG*Δ*ETIT* and *metG630*), as shown graphically in Figure 1. Mutants were generated, as described, and their genotypes confirmed by PCR and DNA sequencing.

### 3.3 Phenotypic characterization of *metG* mutants

**FIGURE 2:**
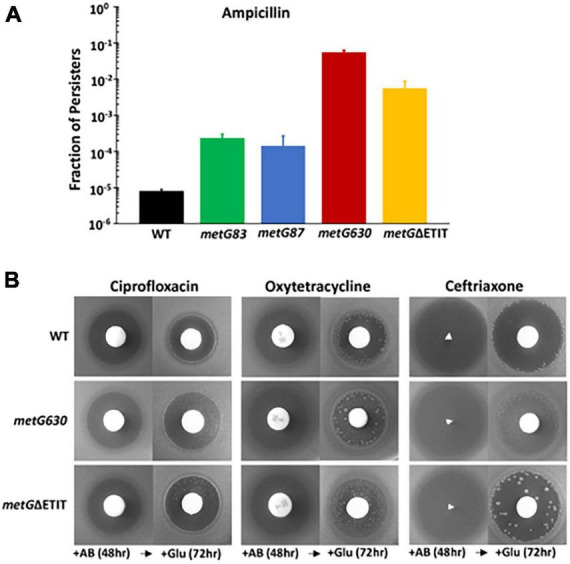
Persistence phenotypes of *metG* mutations in the presence of antibiotics. **(A)** Overnight cultures of wild type and *metG* mutants were assayed for persistence to ampicillin using the penicillinase assay. Persistence was determined as the fraction of persisters in the cultures. **(B)** Qualitative observation of persistence to the 3 antibiotics shown to the two highest persister *metG* mutants as assayed by the tolerance disk test (Gefin). Shown are zones of inhibition against *metG630*, *metG*ΔETIT and wild type strains. Antibiotics were added to filter discs and the plates incubated for 48 h [+AB (48 h)]. The filter discs were then removed and replaced with disks containing glucose followed by incubation for 72 h [+Glu (72 h)]. Shown are the results after 24 h.

To survey the level of persistence for each *metG* mutant, we conducted initial assays as described in see section “2 Materials and methods.” The *metG630* and *metGΔETIT* mutants produced persisters at 2–3 orders of magnitude higher frequency than wild type *E. coli* (Figure 2A). The *metG83* and *metG87* mutants also showed an order of magnitude increase in persistence, indicating that mutations throughout the *metG* coding region can influence the levels of antibiotic persistence. To determine if the *metG* alleles conferred elevated persistence to other antibiotics, we performed plate assays, as described (Gefen et al., 2017). As shown in Figure 2B, both the *metG630* and *metG*Δ*ETIT* mutants showed increased levels of persistence to ciprofloxacin, oxytetracycline, and cephalosporin compared to the wild type control.

Examination of growth kinetics of the *metG* mutants revealed that only the *metG87* mutant exhibited an increase in lag time on rich medium before entering exponential phase (Supplementary Figure 2). *metG87* encodes a S264F replacement near the catalytic site, *metG83* a G263S/S264F double replacement. We concluded that the S264F replacement in *metG* resulted in a slow growing phenotype, and the second G263S replacement found in *metG83* acts to suppress this growth defect. Increased lag times have been reported in persistent and tolerant phenotypes and can be the mechanism behind the persistent phenotype (Fridman et al., 2014; Vulin et al., 2018; Simsek and Kim, 2019). Since we are interested in exploring the effects of MetRS and translation on bacterial antibiotic persistence we excluded *metG87* and focused the rest of our studies on the three *metG* mutants that exhibited similar growth kinetics to WT.

**FIGURE 3:**
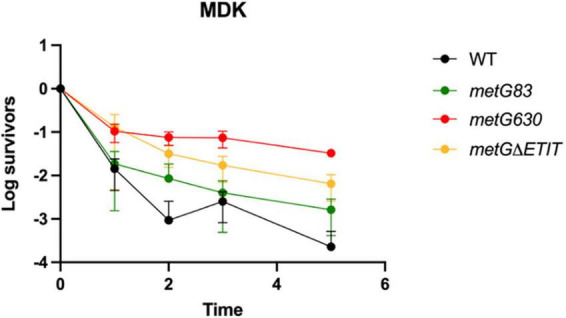
Minimum duration of killing of *metG* mutations in the presence of antibiotics. Minimum duration of killing persistence assays were measured at different hours of exposure to 100 μg/mL ampicillin and represented as survivors on a log scale. *metG83* is a G263S/S264F double mutation near the catalytic site, *metG630* is a I626A frameshift mutation that results in a premature stop codon near the anticodon binding domain, *metG*Δ*ETIT* is a deletion mutation from 569 to 572 near the anticodon binding domain. Error bars represent standard deviations from 3 biological replicates.

Further characterization of the *metG* alleles effects on antibiotic persistence was performed using minimal duration of killing (MDK) assays (Figure 3). The MDK assays showed the characteristic biphasic killing curves that is attributed to bacterial antibiotic persistence (Figure 3; Brauner et al., 2016; Balaban et al., 2019). The rate of killing and the higher number of persisters for the *metG* mutants compared to wild type *E. coli* followed the same pattern as the initial characterization of the *metG* alleles (Figure 2).

### 3.4 Bacterial antibiotic persistence increases when *metG*, but not *alaS*, transcription is knocked down in *E. coli*

We next wanted to investigate if the overall reduction in intracellular concentration of MetRS influenced bacterial antibiotic persistence. We used CRISPRi to reduce the transcript levels of *metG*, which lead to a reduction of intracellular MetRS concentration. Transcription of *alaS*, which encodes for the elongator alanyl-tRNA synthetase, was also knocked down by CRISPRi to ascertain the impact of persistence of an unrelated aaRS (Supplementary Table 2). Growth curves kinetics and colony forming unit experiments were conducted to determine the optimal concentration of anhydrous tetracycline (aTc) that would result in the same knock down efficiency of the *alaS* and *metG* transcripts by the specific small guide RNA (Supplementary Figures 3, 4).

**FIGURE 4:**
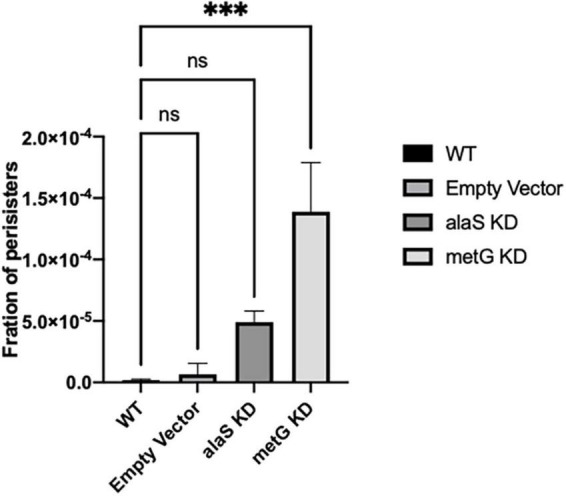
Persistence phenotypes of *E. coli* strains with reduced transcription of *metG* or *alaS*. Bacterial antibiotic endpoint persister assays were performed after 3 h incubation with anhydrous tetracycline to induce CRISPRi knock down of *metG* and *alaS* transcription. The strains were exposed to 100 μg/mL ampicillin overnight. The concentration of anhydrous tetracycline used was determined by growth curves and CFU/mL, 2.0 mg/mL for wild-type and empty vector strains, and 0.5 mg/mL for the *alaS* and *metG* knockdown strains. Empty vector contains no guide RNA. Error bars represent standard deviations from 3 biological replicates. ***p ≤ 0.0001, one-way ANOVA.

Bacterial antibiotic persister endpoint assays were performed to study the effect that *metG* and *alaS* transcription knock down had on persistence (Figure 4). Bacterial antibiotic persistence increased significantly when transcription of *metG* was knocked down compared to wild type *E. coli* and the empty vector (Figure 4). However, when *alaS* was knocked down there was not a significant increase in the level of antibiotic persistence.

### 3.5 Impact of *metG* mutants on protein synthesis

**FIGURE 5:**
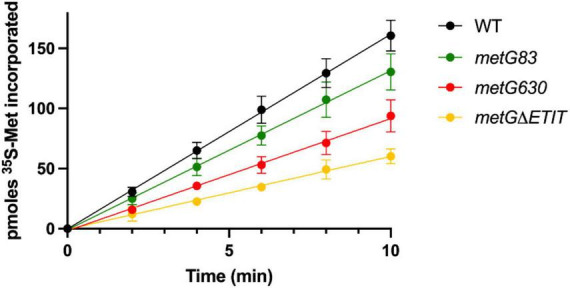
Impact of *metG* mutations on translation rates. The various *metG* mutations were made on the *E. coli* chromosome so that the corresponding MetRS mutation would be made *in vivo*. Cultures were grown in M9 medium supplemented with 50 mM methionine until an OD_600nm_ of 0.7. ^35^S-methionine was added, and measurements were taken every 2 min for 10 min. Error bars represent standard deviations from 3 biological replicates. WT *metG* has a rate of 16.2 pmoles ^35^S-Met/min, *metG83* has a rate of 13.2 pmoles ^35^S-Met/min, *metG630* has a rate of 9.3 pmoles ^35^S-Met/min, and *metG*Δ*ETIT* has a rate of 6.1 pmoles ^35^S-Met/min. All variant metG rates have an *R*^2^ value > 0.99.

We next used pulse-chase labeling to measure rates of protein synthesis of the *metG* mutants in minimal media (Figure 5). In general, slower translation rates, while not sufficient to impact growth rates on complete medium of the *metG* mutants, nevertheless correlated to increases in bacterial antibiotic persistence. This suggests that there may be a connection between translation rates within a cell and its ability to enter a persistent phenotype. To further characterize the translation process, we conducted polysome profiling on each of the mutants (Supplementary Figure 5). The percentage of ribosomes within the 70S fraction was not significantly reduced in three of the mutants (*metG83*, *metG630*, *metG*Δ*ETIT*). These results demonstrate that the *metG* mutants do not have an impact on ribosome assembly but do affect translation rates, consistent with increased disassembled subunits and decreased polysomes. This indicates that the reduced translation rates may be caused by either a defect in translation initiation or the intracellular concentration of methionyl-tRNA*^Met^* if the mutation within MetRS affects its ability to aminoacylate initiator or elongator tRNA*^Met^*, respectively.

**FIGURE 6:**
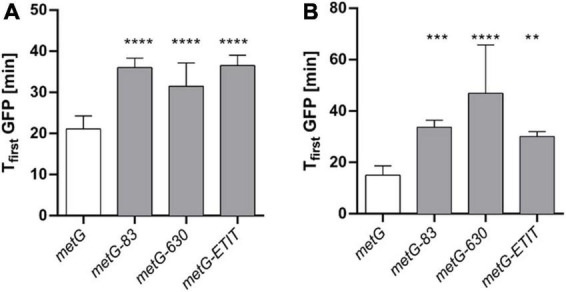
Observation time of GFP in *metG* mutants. A reporter plasmid was used to determine the initial observation times (T_first_ GFP) from the fluorescence intensity kinetics of the GFP plots for each strain. **(A)** Regular GFP and **(B)** a long version of GFP (GFP_Long_) were used to discriminate the most significant effects of *metG* on initiation and elongation. **p ≤ 0.01, ***p ≤ 0.001, ****p ≤ 0.0001, one-way ANOVA with *Dunnett* vs. *metG*.

To investigate if the *metG* mutants primarily affect either the initiation stage of translation or elongation, we used an inducible fluorescent reporter system to evaluate protein synthesis. Under the premise that none of the *metG* mutations affect the rate of transcription or folding of GFP, we assumed that transcription and maturation times are constant for all mutants. By comparing the initial GFP observation time, which corresponds to the minimum time needed to reach the critical mass of fluorescent reporter to be detected, we see an ∼1.5-fold increase in the time required for GFP synthesis for all *metG* mutants (Figure 6A). This supports the results presented previously in Figure 5 in which an impairment in the translation rate was shown. Additionally, a non-fluorescent domain was fused to the N terminus of GFP, nearly doubling the size of the protein and the number of methionines involved in elongation (244 to 477 amino acids and 4 to 8 methionine, respectively) (Figure 6B). The initial observation time for the long GFP variant (GFP_*Long*_) is the same as that of normal-sized GFP for wild type *E. coli*, and the *metG83* and *metG*Δ*ETIT* mutants, demonstrating that the changes generated due to the additional domain that must be elongated do not affect the total translation time. This suggests that while mutations in *metG83* and *metG*Δ*ETIT* mainly affect the initiation step, *metG630* affects both the initiation and elongation steps as evidenced by an increase in the initial observation time for GFP_*Long*_.

### 3.6 Steady-state tRNA aminoacylation kinetics for MetRS variants encoded by *metG* mutants linked to persistence

We next investigated the steady-state kinetics of the MetRS variants to further understand how the different mutations in *metG* affect aminoacylation. Recombinant protein of wild type MetRS and the three variant MetRSs (*metG83*, *metG630*, *metG*Δ*ETIT*) and *in vitro* transcribed elongator tRNA*^Met^* and initator tRNA*^fMet^* was used for steady-state kinetic analyses (Table 1). In general, there was not any significant difference in the aminoacylation kinetics or specific activity of the variant MetRSs when compared to wild type MetRS. These results indicate that the different hyperpersistent mutations found within the *metG* gene do not significantly affect the kinetics for methionine, ATP, tRNA*^Met^*, or tRNA*^fMet^*.

**TABLE 1:** Steady-state aminoacylation kinetics of various MetRS mutations.

K_M_ (μM)	WT	*metG83*	*metG630*	*metG*Δ*ETIT*
Methionine	20.5 ± 3.7	6.2 ± 0.8	11.3 ± 1.0	6.1 ± 1.4
tRNA^Met^	2.2 ± 0.4	1.6 ± 0.3	2.1 ± 0.2	2.5 ± 0.6
tRNA^fMet^	1.9 ± 0.2	0.3 ± 0.1	1.7 ± 0.3	0.9 ± 0.1
ATP	334 ± 58	323 ± 56	252 ± 19	340 ± 8
*K*_*cat*_	7.8 ± 0.4	0.6 ± 0.02	17.3 ± 1.0	7.5 ± 0.2
Specific activity	8.0	1.9	2.9	10.7

Standard deviation is from 3 replicates.

### 3.7 Pre-transfer editing by MetRS variants encoded by *metG* mutants linked to persistence

To further investigate if the MetRS variants have any effect on enzyme chemistry we looked at the pre-transfer editing mechanism. Homocysteine (Hcy) is a non-cognate amino acid for aminoacylation of tRNA*^Met^* by MetRS (Jakubowski, 2003, 2017). If Hcy enters the active site of MetRS it will be pre-transfer edited forming homocysteine thiolactone and releasing tRNA*^Met^*, AMP, and inorganic pyrophosphate (Jakubowski, 2003, 2017). To study the kinetics of the pre-transfer editing mechanism we used recombinant wild type MetRS and three variant MetRSs (*metG83*, *metG630*, *metG*Δ*ETIT*) and *in vitro* transcribed elongator tRNA*^Met^* and initiator tRNA*^fMet^* with three different amino acids, the cognate methionine, and non-cognate homocysteine and S-NO-homocysteine, a natural variant of homocysteine (Jakubowski, 2000). The first step of MetRS’s editing mechanism is to adenylate the amino acid (Ibba and Soll, 2000). Adenylated-homocysteine has a short half-life because the thiol group of the homocysteine is positioned in a way that allows for easy attack of the carboxyl group which releases the more stable metabolite homocysteine thiolactone and AMP (Jakubowski, 2017). However, if homocysteine is modified with a nitrosyl-group it stabilizes the thiol group making adenylated-S-NO-homocysteine more stable than its unmodified amino acid form (Supplementary Figure 6; Jakubowski, 2000, 2003). Therefore, we synthesized S-NO-homocysteine so that we could investigate if MetRS and its mutant variants are able to perform pre-transfer editing of adenylated-homocysteine rather than random hydrolysis of homocysteine to homocysteine thiolactone (Jakubowski, 2000). To study aaRS editing rates we employed ATP futile cycling assays which measure the depletion of ATP to AMP by calculating the amount of P_*i*_ produced (Steiner et al., 2019). As more editing of non-cognate amino acid occurs more ATP is utilized, which in turn produces more inorganic phosphate.

**FIGURE 7:**
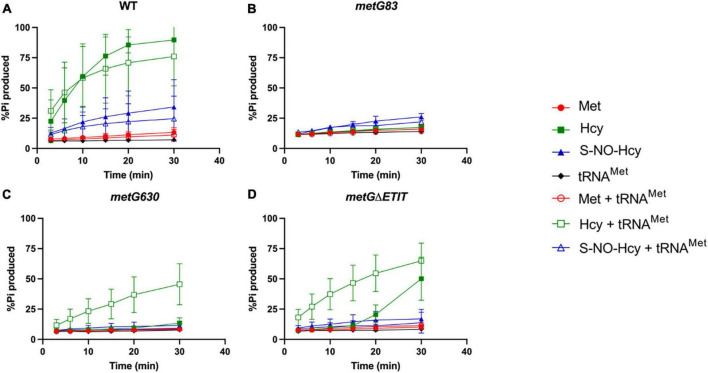
Pre-transfer editing by MetRS variants with elongator tRNA*^Met^*. Pre-transfer editing of L-homocysteine and S-NO-homocysteine was measured as ATP consumed but represented as % P_i_ produced for wild type and variant MetRS mutants with and without tRNA*^Met^*. Time points were taken at 3, 6, 10, 15, 20, and 30 min. **(A)** Wild type MetRS, **(B)** MetRS 83, **(C)** MetRS 630, and **(D)** MetRS ΔETIT. Amino acid with or without tRNA*^Met^* corresponds to key. Error bars represents standard deviations from 3 replicates.

**FIGURE 8:**
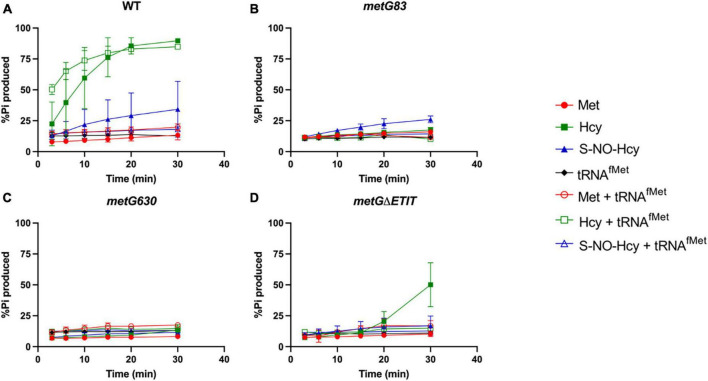
Pre-transfer editing by MetRS variants with initator tRNA*^fMet^*. Pre-transfer editing of L-homocysteine and S-NO-homocysteine was measured as ATP consumed but represented as % P_i_ produced for wild type and variant MetRS mutants with and without tRNA*^fMet^*. Time points were taken at 3, 6, 10, 15, 20, and 30 min. **(A)** Wild type MetRS, **(B)** MetRS 83, **(C)** MetRS 630, and **(D)** MetRS ΔETIT. Amino acid with or without tRNA*^fMet^* corresponds to key. Error bars represents standard deviations from 3 replicates.

We first looked at editing with all three amino acids tested with and without elongator tRNA*^Met^* (Figure 7). As expected, with wild type MetRS we observed no editing with methionine, ∼80–90% editing with homocysteine, and ∼25% editing with S-NO-homocysteine. There was no significant difference in the percentages of editing with and without tRNA*^Met^*, which indicates that wild type MetRS can perform pre-transfer editing with both homocysteine and S-NO-homocysteine. There were no indications of pre-transfer editing with the MetRS 83 variant. Interestingly, MetRS 630 was only able to edit homocysteine once tRNA*^Met^* was added to the reaction. Also, MetRS ΔETIT can edit homocysteine without tRNA*^Met^* present, but its editing activity increases with tRNA*^Met^*. Next, we looked at editing with these three amino acids with and without initiator tRNA*^fMet^* (Figure 8). Intriguingly, there was no editing observed for any of the MetRS variants with initiator tRNA*^fMet^*. Wild type MetRS showed ∼25% editing with S-NO-homocysteine but no editing was observed once tRNA*^fMet^* was added to the reaction. These results indicate that the MetRS variants used in this study have different capabilities of pre-transfer editing.

### 3.8 Growth of variant *E. coli metG* mutants is hypersensitive to homocysteine

**FIGURE 9:**
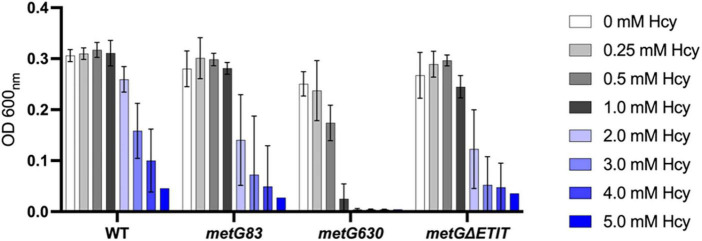
Growth of *E. coli metG* mutants in the presence of homocysteine. Cultures were grown in M9 medium supplemented with 50 mM methionine and various concentrations of homocysteine, ranging from 0 to 5 mM. Growth was measured at OD 600 nm. Error bars represent standard deviations from 3 biological replicates.

Hcy is a key intermediate in central metabolism and necessary for several different pathways such as methionine biosynthesis (Jakubowski and Glowacki, 2011; Ferla and Patrick, 2014). Changes in metabolism and increases in intracellular deacylated-tRNA levels have both been linked to underlying mechanisms of bacterial antibiotic persistence (Amato et al., 2014; Radzikowski et al., 2017; Wood et al., 2021). Since Hcy is important in both metabolism and tRNA*^Met^* aminoacylation we wanted to investigate if Hcy had any effects on the growth of the three different *metG* mutant strains. Overall, all the *metG* mutants (*metG83*, *metG630*, *metG*Δ*ETIT*) were hypersensitive to increasing concentrations of Hcy compared to wild type *E. coli* (Figure 9), suggesting a link between the *metG* mutants, Hcy, and antibiotic persistence.

## 4 Discussion

### 4.1 General defects in translation broadly correlate with bacterial antibiotic persistence

Bacterial translation quality control is important to maintain the homeostasis of the proteome (Bullwinkle and Ibba, 2016; Mohler and Ibba, 2017; Kelly et al., 2019). When translation quality control is absent tRNAs may be misacylated, which can result in protein synthesis errors. This was once thought to be detrimental to the cell, however, it is now understood that altered quality control can sometimes be beneficial as bacterial cells adapt to new environments (Ribas de Pouplana et al., 2014; Rathnayake et al., 2017; Kelly et al., 2019). For example, previous studies using quality control-modified PheRS variants showed that deacylated-tRNA is a trigger for changes in bacterial antibiotic persistence independent of the stringent response (Wood et al., 2021). A genetic screen performed in *E. coli* identified several hyper-persistent mutations in *metG* which encodes for MetRS (Fridman et al., 2014). Specifically, the mutants found were *metG*87, which has a point mutation near the catalytic site (S264F) and *metG*83, a double mutation also near the catalytic site (G263S/S264F). Two additional hyper-persistent mutations that were originally isolated in *S. Typhimurium* were also investigated in this study, *metG630* which is a −4 frameshift mutation resulting in the change of 4 amino acids at the end of a truncated protein near the anticodon binding domain, and *metG*ΔETIT, a deletion mutation removing amino acids 569–572 near the anticodon binding domain (Slattery et al., 2013). Variants in MetRS are of special interest to study how defects in translation can affect bacterial antibiotic persistence because MetRS is responsible for aminoacylation both elongator tRNA*^Met^* and initiator tRNA*^fMet^* (Ibba and Soll, 2000; Laursen et al., 2005). MetRS is also able to perform pre-transfer editing to form homocysteine thiolactone from homocysteine, an important intermediate in central metabolism (Jakubowski, 2003, 2017; Jakubowski and Glowacki, 2011; Ferla and Patrick, 2014). Perturbations in metabolism and consequently in intracellular homocysteine levels could affect the editing activities of MetRS leading to changes in the cellular concentrations of deacylated tRNA*^Met^*. The numerous roles of MetRS in protein synthesis, and indirectly in metabolism, make *metG* mutants of particular interest to investigate bacterial antibiotic persistence.

In our initial characterization of the *metG* mutants, *metG630* and *metG*Δ*ETIT* showed 2–3 orders of magnitude more persisters than wild type *E. coli*, and increased levels of persistence to ciprofloxacin, oxytetracycline, and cephalosporin (Figure 2). The MDK assays of the *metG* mutants showed the characteristic biphasic killing curves and the rate of killing compared to wild type *E. coli* followed the same pattern as the initial characterization of the *metG* alleles (Figure 3). This indicates that mutations throughout *metG* can affect levels of bacterial antibiotic persistence. To see if the increased levels of persistence are linked to defects in translation, we characterized the overall translation rates of the *metG* mutants, along with their polysome profiles. It was observed that slower rates of translation of the *metG* mutants generally correlated with higher levels of antibiotic persistence (Figure 5). However, the 70S ribosome profile fraction was not significantly reduced for the *metG* mutants compared to wild type *E. coli*, indicating that there is not an impact on ribosome assembly but that these mutants do affect overall translation rates. It is worth noting that there are differences amongst the *metG* mutants for the 30S, 50S, and polysome profile fractions. For example, *metG630* has the highest rate of antibiotic persister formation (Figures 2, 3.), the second slowest rate of translation (Figure 5), the highest percentage of 30S and 50S ribosome fractions but the lowest percentage of polysomes (Supplementary Figure 5). This may imply that while there are no differences in the 70S ribosomes the differences in the other fractions of the polysome profile may have a downstream affect that contributes to overall translation defects and bacterial antibiotic persistence increases. This further suggests that the reduced translation rates are caused by a defect in either translation initiation or the intracellular concentration of methionyl-tRNA*^Met^*. However, there were not any significant differences in the aminoacylation kinetics or specific activity of the variant MetRSs when compared to wild type MetRS (Table 1). These results indicate that the different hyperpersistent mutations found within *metG* do not affect the steady-state kinetics of MetRS or its substrates.

Since MetRS is involved in both translation initiation and elongation we further investigated how *metG* mutants may affect these two stages of translation. It was observed that all the *metG* mutants had an increase in time of ∼1.5-fold for translation initiation compared to wild type *E. coli* (Figure 6A). Only the *metG630* mutant also showed an increase in the time for translation elongation when compared to wild type (Figure 6B), consistent with it being the mutant with the largest changes in polysome profile. Overall, the mutants that display the most significant decreases in protein synthesis rates and active translation correlated with higher persistence.

Mutations within the *metG* coding region have a strong correlation between translation defects and bacterial antibiotic persistence, but a poor correlation between MetRS aminoacylation kinetics and persistence. We therefore considered if changes in the intracellular concentration of MetRS affected antibiotic persistence levels or if these effects were due to the changes in the function or activity of MetRS. This was achieved by knocking down the transcription levels of *metG* via CRISPRi technologies. When *metG* was knocked down this resulted in a significant increase in the level of bacterial antibiotic persistence when compared to wild type *E. coli* and an *alaS* knock-down control (Figure 4). These results indicated that some but not all the 20 aminoacyl-tRNA synthetases are implicated in an underlining mechanism of bacterial antibiotic persistence. In a previous study where several PheRS variants were used it was observed that these variants did increase the level of bacterial antibiotic persistence as a result of changes in cellular deacylated tRNA levels (Wood et al., 2021). Reducing the transcription levels of *alaS* produced increased levels of antibiotic persistence broadly comparable to those for PhRS variants; however, the effects on persistence from the *metG* mutations are much stronger, likely due to additive effects on translation initiation.

### 4.2 MetRS variants reveal defects in editing that support increases in bacterial antibiotic persistence

MetRS can perform pre-transfer editing of homocysteine, an important non-proteogenic amino acid that is involved in central metabolism (Jakubowski, 2003, 2017; Jakubowski and Glowacki, 2011; Ferla and Patrick, 2014). The canonical reaction of MetRS is to esterify both tRNA*^Met^* and initiator tRNA*^fMet^* with methionine in an ATP dependent manner (Ibba and Soll, 2000; Laursen et al., 2005). However, if non-cognate homocysteine becomes adenylated, MetRS will perform pre-transfer editing releasing homocysteine thiolactone and deacylated tRNA*^Met/fMet^* (Supplementary Figure 6; Jakubowski, 2003, 2017). A natural modification of homocysteine is S-NO-homocysteine which usually occurs under nitric oxide stress (Braun et al., 2007). S-NO-homocysteine has been detected in human endothelial cells and it has been shown to be able to be translationally incorporated into proteins in *E. coli*, because the S-nitrosyl group stabilizes the thiol group of homocysteine and prevents pre-transfer editing by MetRS (Stamler et al., 1993; Jakubowski, 2000). We studied the kinetics of the pre-transfer editing mechanism of wild type and the three variant MetRSs used in this study along with both elongator tRNA*^Met^* and initator tRNA*^fMet^*. Both tRNAs were *in vitro* transcribed to ensure that they contained their natural first base; tRNA*^Met^* starts with a guanosine base and tRNA*^fMet^* starts with a cytosine base (Laursen et al., 2005). In *E. coli*, tRNA*^fMet^* contains a 1–72 mismatched base pair with is important for the formylation of Met-tRNA*^fMet^* by methionyl-tRNA formyltransferase (Mayer and RajBhandary, 2002; Giege and Eriani, 2023).

As expected, with wild type MetRS and either tRNA*^Met^* or tRNA*^fMet^* there was no editing with methionine observed, ∼80–90% editing with homocysteine, and ∼25% editing with S-NO-homocysteine (Figures 7, 8). There was no editing observed with the MetRS 83 variant with any of the amino acids tested with either tRNA*^Met^* or tRNA*^fMet^*. Intriguingly, the product of the *metRS630* variant was only able to edit homocysteine in the presence of tRNA*^Met^* specifically. MetRSΔETIT was able to modestly edit homocysteine alone, but editing was increased in the presence of tRNA*^Met^* specifically. These MetRS variants have changes to the amino acid sequence near the anti-codon binding domain. This observation may indicate that tRNA*^Met^* binding is required for a tRNA-specific conformational change to induce editing of homocysteine and S-NO-homocysteine. The MetRS630 and MetRSΔETIT variants were unable to edit these non-cognate amino acids in the presence of tRNA*^fMet^*. These results indicate that the MetRS variants used in this study have different capabilities of pre-transfer editing and that the high rate of persistence observed with the *metG* mutant *E. coli* strains are supported by editing deficiencies. The differences observed with initiator tRNA and tRNA*^Met^* in pre-transfer editing also indicate that translation initiation and elongation may be contributing in various ways toward the higher rates of bacterial antibiotic persistence.

### 4.3 Persistent *metG* mutants are highly sensitive to homocysteine

We observed that the different *metG* mutants have increased levels of bacterial antibiotic persistence. These increases may be due to changes in translation initiation and/or translation elongation and the differences that were observed in homocysteine pre-transfer editing activities of the MetRS variants. Since homocysteine is important in both central metabolism and tRNA*^Met^* aminoacylation we investigated if this non-proteogenic amino acid had any effects on the growth of the different *metG* mutant strains used in this study. We observed that all the *metG* mutants were hypersensitive to increasing concentrations of homocysteine compared to wild type *E. coli* (Figure 9). The sensitivity to homocysteine supplementation to growth of the *metG* mutant strains and the defects in editing of the MetRS variants suggest that there is a strong correlation between the *metG* mutants, homocysteine, and antibiotic persistence. Isoleucyl-tRNA synthetase and leucyl-tRNA synthetase are also able to edit homocysteine to homocysteine thiolactone and have both previously been linked to increases in bacterial antibiotic persistence (Garoff et al., 2018; Khare and Tavazoie, 2020). It is interesting to speculate that the levels of homocysteine and homocysteine thiolactone could cause shifts in central metabolism and thus could be an underlining factor contributing to antibiotic persistence.

In conclusion, several different pathways are implicated in the elevated levels of bacterial antibiotic persistence in the *metG* mutant *E. coli* strains used in this study. We observed defects in translation initiation and elongation rates, different defects in the editing capabilities of the MetRS variants, and hyper-sensitivity to the growth of the *metG* mutant *E. coli* strains when supplemented with homocysteine. Intriguingly, all these changes in the *E. coli* cell could have a compounding effect resulting in the high levels of bacterial antibiotic persistence that were observed. Several aaRSs have been found to impact the levels of bacterial antibiotic persistence (Germain et al., 2013; Kaspy et al., 2013; Levin-Reisman et al., 2017; Garoff et al., 2018; Hatch and Ouellette, 2020; Huang et al., 2020; Khare and Tavazoie, 2020; Wood et al., 2021). However, any contributions from translation initiation to the increased levels of antibiotic persistence would be specific to MetRS. Taken together, these data suggest that while there is a general translation effect, as with many aaRSs, the MetRS variants have a stronger effect on antibiotic persistence that may be due to their editing defects and the resulting changes in cellular homocysteine and homocysteine thiolactone levels.

## Data availability statement

The original contributions presented in this study are included in the article/Supplementary material, further inquiries can be directed to the corresponding author.

## Author contributions

WW: Conceptualization, Data curation, Formal analysis, Investigation, Supervision, Writing – original draft, Writing – review and editing, Methodology, Validation, Visualization. MR: Conceptualization, Data curation, Formal analysis, Investigation, Methodology, Validation, Visualization, Writing – review and editing. LL: Data curation, Formal analysis, Investigation, Methodology, Validation, Visualization, Writing – review and editing. GP: Data curation, Formal analysis, Investigation, Methodology, Validation, Visualization, Writing – review and editing, Conceptualization, Funding acquisition, Project administration, Resources, Supervision. MI: Conceptualization, Data curation, Formal analysis, Funding acquisition, Investigation, Project administration, Resources, Supervision, Writing – review and editing, Writing – original draft.

## References

[B1] Amato S. M. Fazen C. H. Henry T. C. Mok W. W. Orman M. A. Sandvik E. L. (2014). The role of metabolism in bacterial persistence. *Front. Microbiol.* 5:70. 10.3389/fmicb.2014.00070 24624123 PMC3939429

[B2] Baba T. Ara T. Hasegawa M. Takai Y. Okumura Y. Baba M. (2006). Construction of *Escherichia coli* K-12 in-frame, single-gene knockout mutants: The Keio collection. *Mol. Syst. Biol.* 2:0008. 10.1038/msb4100050 16738554 PMC1681482

[B3] Backes N. Phillips G. J. (2021). Repurposing CRISPR-Cas systems as genetic tools for the *Enterobacteriales*. *EcoSal Plus* 9:eES00062020. 10.1128/ecosalplus.ESP-0006-2020 34125584 PMC11163844

[B4] Balaban N. Q. Gerdes K. Lewis K. Mckinney J. D. (2013). A problem of persistence: Still more questions than answers? *Nat. Rev. Microbiol.* 11 587–591. 24020075 10.1038/nrmicro3076

[B5] Balaban N. Q. Helaine S. Lewis K. Ackermann M. Aldridge B. Andersson D. I. (2019). Definitions and guidelines for research on antibiotic persistence. *Nat. Rev. Microbiol.* 17 441–448. 30980069 10.1038/s41579-019-0196-3 PMC7136161

[B6] Blumenthal T. (1972). P1 transduction: Formation of heterogenotes upon cotransduction of bacterial genes with a P2 prophage. *Virology* 47 76–93. 10.1016/0042-6822(72)90241-3 4550792

[B7] Braun O. Knipp M. Chesnov S. Vasak M. (2007). Specific reactions of S-nitrosothiols with cysteine hydrolases: A comparative study between dimethylargininase-1 and CTP synthetase. *Protein Sci.* 16 1522–1534. 10.1110/ps.062718507 17600152 PMC2203367

[B8] Brauner A. Fridman O. Gefen O. Balaban N. Q. (2016). Distinguishing between resistance, tolerance and persistence to antibiotic treatment. *Nat. Rev. Microbiol.* 14 320–330. 27080241 10.1038/nrmicro.2016.34

[B9] Bullwinkle T. J. Ibba M. (2016). Translation quality control is critical for bacterial responses to amino acid stress. *Proc. Natl. Acad. Sci. U.S.A.* 113 2252–2257. 26858451 10.1073/pnas.1525206113 PMC4776511

[B10] Casini A. Macdonald J. T. de Jonghe J. Christodoulou G. Freemont P. S. Baldwin G. S. (2014). One-pot DNA construction for synthetic biology: The Modular overlap-directed assembly with linkers (MODAL) strategy. *Nucleic Acids Res.* 42:e7. 10.1093/nar/gkt915 24153110 PMC3874208

[B11] Chung C. T. Niemela S. L. Miller R. H. (1989). One-step preparation of competent *Escherichia coli*: Transformation and storage of bacterial cells in the same solution. *Proc. Natl. Acad. Sci. U.S.A.* 86 2172–2175. 10.1073/pnas.86.7.2172 2648393 PMC286873

[B12] Cotten K. L. Davis K. M. (2023). Bacterial heterogeneity and antibiotic persistence: Bacterial mechanisms utilized in the host environment. *Microbiol. Mol. Biol. Rev.* 87:e0017422. 10.1128/mmbr.00174-22 PMC10732018 37962348

[B13] Cui L. Vigouroux A. Rousset F. Varet H. Khanna V. Bikard D. (2018). A CRISPRi screen in *E. coli* reveals sequence-specific toxicity of dCas9. *Nat. Commun.* 9:1912. 10.1038/s41467-018-04209-5 29765036 PMC5954155

[B14] Dardel F. Fayat G. Blanquet S. (1984). Molecular cloning and primary structure of the *Escherichia coli* methionyl-tRNA synthetase gene. *J. Bacteriol.* 160 1115–1122. 10.1128/jb.160.3.1115-1122.1984 6094501 PMC215828

[B15] Datsenko K. A. Wanner B. L. (2000). One-step inactivation of chromosomal genes in *Escherichia coli* K-12 using PCR products. *Proc. Natl. Acad. Sci. U.S.A.* 97 6640–6645. 10.1073/pnas.120163297 10829079 PMC18686

[B16] Fechter P. Rudinger J. Giege R. Theobald-Dietrich A. (1998). Ribozyme processed tRNA transcripts with unfriendly internal promoter for T7 RNA polymerase: Production and activity. *FEBS Lett.* 436 99–103. 10.1016/s0014-5793(98)01096-5 9771901

[B17] Ferla M. P. Patrick W. M. (2014). Bacterial methionine biosynthesis. *Microbiology (Reading)* 160 1571–1584. 24939187 10.1099/mic.0.077826-0

[B18] Ferrieres L. Hemery G. Nham T. Guerout A. M. Mazel D. Beloin C. (2010). Silent mischief: Bacteriophage Mu insertions contaminate products of *Escherichia coli* random mutagenesis performed using suicidal transposon delivery plasmids mobilized by broad-host-range RP4 conjugative machinery. *J. Bacteriol.* 192 6418–6427. 10.1128/JB.00621-10 20935093 PMC3008518

[B19] Fisher R. A. Gollan B. Helaine S. (2017). Persistent bacterial infections and persister cells. *Nat. Rev. Microbiol.* 15 453–464. 28529326 10.1038/nrmicro.2017.42

[B20] Francklyn C. S. First E. A. Perona J. J. Hou Y. M. (2008). Methods for kinetic and thermodynamic analysis of aminoacyl-tRNA synthetases. *Methods* 44 100–118. 18241792 10.1016/j.ymeth.2007.09.007 PMC2288706

[B21] Fridman O. Goldberg A. Ronin I. Shoresh N. Balaban N. Q. (2014). Optimization of lag time underlies antibiotic tolerance in evolved bacterial populations. *Nature* 513 418–421. 10.1038/nature13469 25043002

[B22] Garoff L. Huseby D. L. Praski Alzrigat L. Hughes D. (2018). Effect of aminoacyl-tRNA synthetase mutations on susceptibility to ciprofloxacin in *Escherichia coli*. *J. Antimicrob. Chemother.* 73 3285–3292. 10.1093/jac/dky356 30239743

[B23] Gefen O. Chekol B. Strahilevitz J. Balaban N. Q. (2017). TDtest: Easy detection of bacterial tolerance and persistence in clinical isolates by a modified disk-diffusion assay. *Sci. Rep.* 7:41284. 10.1038/srep41284 28145464 PMC5286521

[B24] Germain E. Castro-Roa D. Zenkin N. Gerdes K. (2013). Molecular mechanism of bacterial persistence by HipA. *Mol. Cell* 52 248–254. 24095282 10.1016/j.molcel.2013.08.045

[B25] Giege R. Eriani G. (2023). The tRNA identity landscape for aminoacylation and beyond. *Nucleic Acids Res.* 51 1528–1570. 10.1093/nar/gkad007 36744444 PMC9976931

[B26] Giege R. Springer M. (2016). Aminoacyl-tRNA synthetases in the bacterial world. *EcoSal Plus* 7:18. 10.1128/ecosalplus.esp-0002-2016 PMC11575706 27223819

[B27] Girgis H. S. Harris K. Tavazoie S. (2012). Large mutational target size for rapid emergence of bacterial persistence. *Proc. Natl. Acad. Sci. U.S.A.* 109 12740–12745. 22802628 10.1073/pnas.1205124109 PMC3411964

[B28] Greene R. C. Hunter J. S. Coch E. H. (1973). Properties of metK mutants of *Escherichia coli* K-12. *J. Bacteriol.* 115 57–67. 10.1128/jb.115.1.57-67.1973 4577753 PMC246212

[B29] Harms A. Maisonneuve E. Gerdes K. (2016). Mechanisms of bacterial persistence during stress and antibiotic exposure. *Science* 354:aaf4268. 10.1126/science.aaf4268 27980159

[B30] Hatch N. Ouellette S. P. (2020). Inhibition of tRNA synthetases induces persistence in chlamydia. *Infect. Immun.* 88:e00943–19. 10.1128/IAI.00943-19 31964747 PMC7093126

[B31] Huang C. Y. Gonzalez-Lopez C. Henry C. Mijakovic I. Ryan K. R. (2020). hipBA toxin-antitoxin systems mediate persistence in *Caulobacter crescentus*. *Sci. Rep.* 10:2865. 10.1038/s41598-020-59283-x 32071324 PMC7029023

[B32] Ibba M. Soll D. (2000). Aminoacyt-tRNA synthetases. *Annu. Rev. Biochem.* 26 617–650. 10.1146/annurev.biochem.69.1.617 10966471

[B33] Ibba M. Kast P. Hennecke H. (1994). Substrate specificity is determined by amino acid binding pocket size in *Escherichia coli* phenylalanyl-tRNA synthetase. *Biochemistry* 33 7107–7112. 10.1021/bi00189a013 8003476

[B34] Jakubowski H. (2000). Translational incorporation of S-nitrosohomocysteine into protein. *J. Biol. Chem.* 275 21813–21816. 10.1074/jbc.C000280200 10829011

[B35] Jakubowski H. (2003). Homocysteine-thiolactone and S-nitroso-homocysteine mediate incorporation of homocysteine into protein in humans. *Clin. Chem. Lab. Med.* 41 1462–1466. 10.1515/CCLM.2003.224 14656026

[B36] Jakubowski H. (2017). Homocysteine editing, thioester chemistry, coenzyme a, and the origin of coded peptide synthesis dagger. *Life (Basel)* 7:6. 10.3390/life7010006 28208756 PMC5370406

[B37] Jakubowski H. Glowacki R. (2011). Chemical biology of homocysteine thiolactone and related metabolites. *Adv. Clin. Chem.* 55 81–103. 22126025 10.1016/b978-0-12-387042-1.00005-8

[B38] Kalogeraki V. S. Winans S. C. (1997). Suicide plasmids containing promoterless reporter genes can simultaneously disrupt and create fusions to target genes of diverse bacteria. *Gene* 188 69–75. 10.1016/s0378-1119(96)00778-0 9099861

[B39] Kaspy I. Rotem E. Weiss N. Ronin I. Balaban N. Q. Glaser G. (2013). HipA-mediated antibiotic persistence via phosphorylation of the glutamyl-tRNA-synthetase. *Nat. Commun.* 4:3001. 10.1038/ncomms4001 24343429

[B40] Kelly P. Backes N. Mohler K. Buser C. Kavoor A. Rinehart J. (2019). Alanyl-tRNA synthetase quality control prevents global dysregulation of the *Escherichia coli* proteome. *mBio* 10:1222. 10.1128/mBio.02921-19 31848288 PMC6918089

[B41] Khare A. Tavazoie S. (2020). Extreme antibiotic persistence via heterogeneity-generating mutations trageting translation. *mSystems* 5:e00847–19. 10.1128/mSystems.00847-19 31964772 PMC6977076

[B42] Kim J. Webb A. M. Kershner J. P. Blaskowski S. Copley S. D. (2014). A versatile and highly efficient method for scarless genome editing in *Escherichia coli* and *Salmonella enterica*. *BMC Biotechnol.* 14:84. 10.1186/1472-6750-14-84 25255806 PMC4236582

[B43] Kwan B. W. Valenta J. A. Benedik M. J. Wood T. K. (2013). Arrested protein synthesis increases persister-like cell formation. *Antimicrob. Agents Chemother.* 57 1468–1473. 10.1128/AAC.02135-12 23295927 PMC3591907

[B44] Laursen B. S. Sorensen H. P. Mortensen K. K. Sperling-Petersen H. U. (2005). Initiation of protein synthesis in bacteria. *Microbiol. Mol. Biol. Rev.* 69 101–123. 15755955 10.1128/MMBR.69.1.101-123.2005 PMC1082788

[B45] Lee D. J. Bingle L. E. Heurlier K. Pallen M. J. Penn C. W. Busby S. J. (2009). Gene doctoring: A method for recombineering in laboratory and pathogenic *Escherichia coli* strains. *BMC Microbiol.* 9:252. 10.1186/1471-2180-9-252 20003185 PMC2796669

[B46] Leiva L. E. Elgamal S. Leidel S. A. Orellana O. Ibba M. Katz A. (2022). Oxidative stress strongly restricts the effect of codon choice on the efficiency of protein synthesis in *Escherichia coli*. *Front. Microbiol.* 13:1042675. 10.3389/fmicb.2022.1042675 36532460 PMC9749903

[B47] Levin-Reisman I. Brauner A. Ronin I. Balaban N. Q. (2019). Epistasis between antibiotic tolerance, persistence, and resistance mutations. *Proc. Natl. Acad. Sci. U.S.A.* 116 14734–14739. 10.1073/pnas.1906169116 31262806 PMC6642377

[B48] Levin-Reisman I. Ronin I. Gefen O. Braniss I. Shoresh N. Balaban N. Q. (2017). Antibiotic tolerance facilitates the evolution of resistance. *Science* 355 826–830. 28183996 10.1126/science.aaj2191

[B49] Lewis K. (2007). Persister cells, dormancy and infectious disease. *Nat. Rev. Microbiol.* 5 48–56. 17143318 10.1038/nrmicro1557

[B50] Mayer C. RajBhandary U. L. (2002). Conformational change of *Escherichia coli* initiator methionyl-tRNA(fMet) upon binding to methionyl-tRNA formyl transferase. *Nucleic Acids Res.* 30 2844–2850. 10.1093/nar/gkf411 12087168 PMC117066

[B51] Mohler K. Ibba M. (2017). Translational fidelity and mistranslation in the cellular response to stress. *Nat. Microbiol.* 2:17117. 10.1038/nmicrobiol.2017.117 PMC5697424 28836574

[B52] Mohler K. Mann R. Kyle A. Reynolds N. Ibba M. (2018). Aminoacyl-tRNA quality control is required for efficient activation of the TOR pathway regulator Gln3p. *RNA Biol.* 15 594–603. 10.1080/15476286.2017.1379635 28910581 PMC6103723

[B53] Oster C. J. Phillips G. J. (2011). Vectors for ligation-independent construction of lacZ gene fusions and cloning of PCR products using a nicking endonuclease. *Plasmid* 66 180–185. 10.1016/j.plasmid.2011.07.007 21854804 PMC3220417

[B54] Pang L. Weeks S. D. van Aerschot A. (2021). Aminoacyl-tRNA synthetases as valuable targets for antimicrobial drug discovery. *Int. J. Mol. Sci.* 22:1750. 10.3390/ijms22041750 PMC7916415 33578647

[B55] Phillips G. (2004). “Plasmids as genetic tools for study of bacterial gene function,” in *Plasmid biology*, eds Funnell B. E. Phillips G. (Hoboken, NJ: Wiley).

[B56] Phillips G. J. Park S. K. Huber D. (2000). High copy number plasmids compatible with commonly used cloning vectors. *Biotechniques* 28 400–402. 10723548 10.2144/00283bm02

[B57] Posfai G. Kolisnychenko V. Bereczki Z. Blattner F. R. (1999). Markerless gene replacement in *Escherichia coli* stimulated by a double-strand break in the chromosome. *Nucleic Acids Res.* 27 4409–4415. 10.1093/nar/27.22.4409 10536150 PMC148724

[B58] Qi L. S. Larson M. H. Gilbert L. A. Doudna J. A. Weissman J. S. Arkin A. P. (2013). Repurposing CRISPR as an RNA-guided platform for sequence-specific control of gene expression. *Cell* 152 1173–1183. 23452860 10.1016/j.cell.2013.02.022 PMC3664290

[B59] Qin D. Fredrick K. (2013). Analysis of polysomes from bacteria. *Methods Enzymol.* 530 159–172. 24034320 10.1016/B978-0-12-420037-1.00008-7

[B60] Radzikowski J. L. Schramke H. Heinemann M. (2017). Bacterial persistence from a system-level perspective. *Curr. Opin. Biotechnol.* 46 98–105. 28292710 10.1016/j.copbio.2017.02.012

[B61] Rathnayake U. M. Wood W. N. Hendrickson T. L. (2017). Indirect tRNA aminoacylation during accurate translation and phenotypic mistranslation. *Curr. Opin. Chem. Biol.* 41 114–122. 10.1016/j.cbpa.2017.10.009 29156229

[B62] Ribas de Pouplana L. Santos M. A. Zhu J. H. Farabaugh P. J. Javid B. (2014). Protein mistranslation: Friend or foe? *Trends Biochem. Sci.* 39 355–362. 25023410 10.1016/j.tibs.2014.06.002

[B63] Roy H. Ling J. Alfonzo J. Ibba M. (2005). Loss of editing activity during the evolution of mitochondrial phenylalanyl-tRNA synthetase. *J. Biol. Chem.* 280 38186–38192. 10.1074/jbc.M508281200 16162501

[B64] Schleif R. Hess W. Finkelstein S. Ellis D. (1973). Induction kinetics of the L-arabinose operon of *Escherichia coli*. *J. Bacteriol.* 115 9–14. 4577756 10.1128/jb.115.1.9-14.1973 PMC246203

[B65] Silva-Valenzuela C. A. Lazinski D. W. Kahne S. C. Nguyen Y. Molina-Quiroz R. C. Camilli A. (2017). Growth arrest and a persister state enable resistance to osmotic shock and facilitate dissemination of *Vibrio cholerae*. *ISME J.* 11 2718–2728. 10.1038/ismej.2017.121 28742070 PMC5702728

[B66] Simsek E. Kim M. (2019). Power-law tail in lag time distribution underlies bacterial persistence. *Proc. Natl. Acad. Sci. U.S.A.* 116 17635–17640. 10.1073/pnas.1903836116 31427535 PMC6731627

[B67] Slattery A. Victorsen A. H. Brown A. Hillman K. Phillips G. J. (2013). Isolation of highly persistent mutants of *Salmonella enterica* serovar typhimurium reveals a new toxin-antitoxin module. *J. Bacteriol.* 195 647–657. 10.1128/JB.01397-12 23204462 PMC3562109

[B68] Stamler J. S. Osborne J. A. Jaraki O. Rabbani L. E. Mullins M. Singel D. (1993). Adverse vascular effects of homocysteine are modulated by endothelium-derived relaxing factor and related oxides of nitrogen. *J. Clin. Invest.* 91 308–318. 10.1172/JCI116187 8380812 PMC330028

[B69] Steiner R. E. Kyle A. M. Ibba M. (2019). Oxidation of phenylalanyl-tRNA synthetase positively regulates translational quality control. *Proc. Natl. Acad. Sci. U.S.A.* 116 10058–10063. 10.1073/pnas.1901634116 31036643 PMC6525502

[B70] van den Bergh B. Schramke H. Michiels J. E. Kimkes T. E. P. Radzikowski J. L. Schimpf J. (2022). Mutations in respiratory complex I promote antibiotic persistence through alterations in intracellular acidity and protein synthesis. *Nat. Commun.* 13:546. 10.1038/s41467-022-28141-x 35087069 PMC8795404

[B71] Vulin C. Leimer N. Huemer M. Ackermann M. Zinkernagel A. S. (2018). Prolonged bacterial lag time results in small colony variants that represent a sub-population of persisters. *Nat. Commun.* 9:4074. 10.1038/s41467-018-06527-0 30287875 PMC6172231

[B72] Wong S. M. Mekalanos J. J. (2000). Genetic footprinting with mariner-based transposition in *Pseudomonas aeruginosa*. *Proc. Natl. Acad. Sci. U.S.A.* 97 10191–10196. 10.1073/pnas.97.18.10191 10963681 PMC27802

[B73] Wood W. N. Mohler K. Rinehart J. Ibba M. (2021). Deacylated tRNA accumulation is a trigger for bacterial antibiotic persistence independent of the stringent response. *mBio* 12:e0113221. 10.1128/mBio.01132-21 34126764 PMC8262941

[B74] Yi H. Lee H. Cho K. H. Kim H. S. (2018). Mutations in MetG (methionyl-tRNA synthetase) and TrmD [tRNA (guanine-N1)-methyltransferase] conferring meropenem tolerance in *Burkholderia thailandensis*. *J. Antimicrob. Chemother.* 73 332–338. 29136176 10.1093/jac/dkx378

[B75] Yu D. Ellis H. M. Lee E. C. Jenkins N. A. Copeland N. G. Court D. L. (2000). An efficient recombination system for chromosome engineering in *Escherichia coli*. *Proc. Natl. Acad. Sci. U.S.A.* 97 5978–5983. 10.1073/pnas.100127597 10811905 PMC18544

[B76] Zhu Z. Zhou X. Li B. Wang S. Cheng F. Zhang J. (2018). Genomic analysis and resistance mechanisms in *Shigella flexneri* 2a strain 301. *Microb. Drug Resist.* 24 323–336. 10.1089/mdr.2016.0173 28853989

