# Supplementary material for: Methionyl-tRNA synthetase synthetic and proofreading activities are determinants of antibiotic persistence
Source: Front Microbiol. 2024 Mar 27;15:1384552. doi: 10.3389/fmicb.2024.1384552 (PMC11004401; doi:10.3389/fmicb.2024.1384552)

# Supplementary Information

## RESULTS

### Base pair substitutions and deletions of the *lac* operon

To test the efficiency of the RIPR system for gene editing, we first conducted several genome edits of the *lac* operon of *E. coli*. For this, we targeted the nucleotides that encode amino acid position 461, which is required for β-galactosidase activity, and allowing visual screening of *lacZ* mutants by addition of X-gal to the selective media
(Cupples and Miller, 1988). As described in Materials and Methods, we first generated 1100-bp PCR products representing *lacZ* using genomic DNA from strains CSH101 (*lacZ571,* E461Am) and CSH102 (*lacZ572,* E461G) as templates (SI Table 1). The PCR products were cloned into pRIPR and the resulting plasmids introduced into a wild type *E. coli* K-12 strain by conjugation, as described in Materials and Methods. Recombinants were isolated following expression of the ISce-I meganuclease and tested on LB plates with X-gal. As shown in Figure 1, introduction of an amber nonsense mutation (*lacZ572*), originally form CSH101, by RIPR yielded mutants with complete loss of β-galactosidase activity, while introduction of *lacZ572* from CSH102 resulted in partial enzyme activity. These phenotypes are consistent with those reported previously for β-galactosidase (Cupples and Miller, 1988).

Similar results were obtained using RIPR to delete *lacZ*. SI Figure 1 shows that upon plating recombinants directly onto X-gal plates, *lacZ* deletion mutants in roughly 50% of the colonies, which reflected the predicted phenotype of the specific mutations (SI Table 3). DNA sequencing of PCR products confirmed that the predicted base pair changes were made (Cupples and Miller, 1988).

We next tested the ability of RIPR to delete the entire *lac* operon from *E. coli*. For this, we took advantage of affordable synthetic DNA fragments to construct a pRIPR vector with 500-bp of homology (1000-bp total) to the chromosome flanking the *lac* operon (SI Figure 1). We also constructed a second pRIPR derivative with 500-bp synthetic DNA fragment with 250-bp of homology flanking *lac*, which is near the lower limit for efficient Rec-dependent recombination (Lovett et al., 2002). Interestingly, the pRIPR construct with longer region of homology did not yield more recombinants, although testing a larger number of colonies may reveal differences in allelic exchange frequency (SI Table 3). Regardless, deletion of the entire *lac* operon was confirmed by PCR and DNA sequencing at high efficiency using as short as 250-bp of flanking DNA homology.

**SI TABLE 1. *Escherichia coli* strains and plasmids used in this study.**

| Strain or plasmid | Relevant Features | Source or Reference |
|---|---|---|
| Strains | | |
| NEB5a™ | *fhuA2,* Δ(*argF-lacZ*)U169, *phoA*, *glnV44,* Φ80Δ(*lacZ*)M15, *gyrA96*, *recA1*, *relA1*, *endA1*, *thi-1*, *hsdR17* | New England Biolabs |
| EC100D™*pir*+ | F - *mcr*A Δ(*mrr-hsdRMS-mcrBC*) φ80d*lacZ*ΔM15 Δ*lacX74 recA*1 *endA*1 *araD139* Δ(*ara*, *leu*)7697 *galU galK* λ- *rpsL* (StrR) *nupG pir*+. Host strain for pRIPR plasmids. | LGC Biosearch Technologies, Inc. |
| MFD*pir* | MG1655 RP4-2-Tc::[ΔMu1::*aac*(3)IV-Δ*aphA*-Δ*nic35*-ΔMu2::*zeo*] Δ*dapA*::(*erm-pir*) Δ*recA*. Donor strain for plasmid conjugation. | (Ferrieres et al., 2010) |
| BW30270 | MG1655 *rph*$^+$ (wild-type strain) | Coli Genetic Stock Center |
| LC-E75 | MG1655, I *attB:*pOSIP-KL-mCherry, 186 *attB:* pOSIP-CO-RBS-library dCas9 (2-3) | (Cui et al., 2018) |
| CSH101 | *araA*, D(*gpt-lac)5*, *relA1*, *spoT1, thiE1*, F'128, *lacI373*, *lacZ571(Am)* | Coli Genetic Stock Center (Cupples and Miller, 1989) |
| CSH102 | *araA*, D(*gpt-lac)5*, *relA1*, *spoT1, thiE1*, F'128, *lacI373*, *lacZ572* | Coli Genetic Stock Center (Cupples and Miller, 1989) |
| NB116 | BW30270 *ΔlacIZYA* | This study |
| NB129 | BW30270 *metG630* (*-4TGAT2183*) | This study |
| NB134 | BW30270 *metG83* (G263S, S264F) | This Study (Blumenthal, 1972) |
| NB143 | BW30270 *metG87* (S264F) | This study (Greene et al., 1973) |
| NB162 | BW30270 *metGΔETIT* (*ΔGAAACCATCACC2011*) | This study, (Levin-Reisman et al., 2017) |
| Plasmids | | |
| pRIPR | *oriT*$_{RP4}$, ori$_{R6K}$, Cam$^R$, Kan$^R$, | This study |
| pSceH | SC101*ori*$^{TS}$, P$_{tet}$, *I-SceI*, Amp$^R$ | This study (Kim et al., 2014) |
| pDLC-sgRNA | RSF1030*ori*, Cam$^R$ | This study (Phillips et al., 2000) |

**SI TABLE 2. Primers and synthetic DNA fragments used in this study.**

| DNA | Sequence (5’-3’) |
| --- | --- |
| Primers | |
| VIKdel.S | Phos-CGCAAAGAGAAAGCAGGTAG |
| VIKdel.AS | Phos- GGTAGTTCAGGCAGTTCAATC |
| KD3-Sce-SacI.S | GAGCTCTAGGGATAACAGGGTAATTGTGTAGGCTGGAGCTGCTTC |
| KD3-Sce-SacI.AS | GAGCTCATATGAATATCCTCCTTAG |
| VIK-NheI.S | AAGCTAGCTCTATGAAAGGTTGGGCTT |
| VIK-NheI.AS | AAGCTAGCTTACTTTGCAGGGCTTCC |
| BspQI-KD4.S | GCTAGCTCTTCCTCGACATTCCAAGGCCTGTGTAGGCTGGAGCTGCTT C |
| BspQI-KD4.AS | GCTAGCTCTTCAACTCATCGACTAGGCCTCATATGAATATCCTCCTTAG |
| BspQI-lacIZYA-1.S | TCGACATTCCAAGGGGAAGAGCCAGCCGCTTATCCTTTC |
| BspQI-lacIZYA-1.AS | ACTCATCGACTAGGTGAAGAGCCTTTGCACCAGTACGTTT |
| BspQI-lacIZYA-2.S | TCGACATTCCAAGGGGAAGAGCACACCATCGAATGGCGCAAAACCC |
| BspQI-lacIZYA-2.AS | ACTCATCGACTAGGTGAAGAGCCATAAGCGCAGCGTATCAG |
| BspQI-lacZ.S | TCGACATTCCAAGGGGAAGAGCCCGAAATCCCGAATCTCTATC |
| BspQI-lacZ.AS | ACTCATCGACTAGGTGAAGAGCCACATCCAGAGGCACTTCAC |
| BspQI-metG.S | TCGACATTCCAAGGGGAAGAGCTCGTTCATCCCGACTCCTTT |
| BspQI-metG.AS | ACTCATCGACTAGGTGAAGAGCCCGATGCAGCGCCTTGAA |
| BspQI-metG630.S | TCGACATTCCAAGGGGAAGAGCTGCTCAATGGGCATCAAC |
| BspQI-metG630.AS | ACTCATCGACTAGGTGAAGAGCCATCCCTAGCTTGCAAAGAATTA |
| BspQI-metG-ETIT.S | TCGACATTCCAAGGGGAAGAGCATGCCGCTGAAGTGATTG |

| | |
|---|---|
| BspQI-metG-ETIT.AS | ACTCATCGACTAGGTGAAGAGCCTCAACTGCCCTGAATTTATGT |
| BspQI-metG.S | TCGACATTCCAAGGGGAAGAGC TCGTTCATCCCGACTCCTTT |
| BspQI-metG.AS | ACTCATCGACTAGGTGAAGAGCCCGATGCAGCGCCTTGAA |
| pgRNA-alaS.S | GCTTGATTTCGTTTTAGAGCTAGAAATAGCAAGTTAAAATAAGG |
| pgRNA-alaS.AS | TAACGAAATAAGACCGCTAAACTGAAAGTTACTAGTATTATAC |
| pgRNA-metG.S | TTTTCCCATTGTTTTAGAGCTAGAAATAGCAAGTTAAAATAAGG |
| pgRNA-metG.AS | TGTTAAGTAA AGACCGCTAAACTGAAAGTTACTAGTATTATAC |
| gBlocks | |
| D-lacIZYA-1 | CAGCCGCTTATCCTTTCACCGGGCAATGGTCGGGCGACGTTTGCCGCTTCTGAAAACCGCCTCGGGCCTGACCTGGCTGGCCTTTTGCCCGGAACAAGACCGCAAGGAATTAATCGAAATGTTAGCCTCCCGCCCCGGTGATGACTATCAACTGGCACGGGAACCGTTAAAGCTGGAAGCCATTCTGGCGCGCGCGCGCAAAGAGGGTTACGGACAGAACTACCGCGGCTGGGATCAGGAGGAGAAGATCGCCTCTATCGCCGTACCGCTGCGCAGTGAACAACGGGTGATTGGCTGTCTGAATCTGGTGTATATGGCGAGCGCAATGACCATTGAACAGGCAGCGGAAAAGCATCTTCCGGCGCTACAACGGGTAGCAAAACAGATCGAAGAAGGGGTTGAATCGCAGGCTATTCTGGTGGCCGGAAGGCGAAGCGGCATGCATTTACGTTGACCCAACGTTACTCTTTCCGTTACGGGACACCCTGTACACCATGAATTGAGAAAAAACGGCGAGATGTACTCTTTTCCGATAACGATTGGCAATAACGTCTGGATCGGAAGTCATGTGGTTATTAATCCAGGCGTCACCATCGGGGATAATTCTGTTATTGGCGCGGGTAGTATCGTCACAAAAGACATTCCACCAAACGTCGTGGCGGCTGGCGTTCCTTGTCGGGTTATTCGCGAAATAAACGACCGGGATAAGCACTATTATTTCAAAGATTATAAAGTTGAATCGTCAGTTTAAATTATAAAAATTGCCTGATACGCTGCGCTTATCAGGCCTACAAGTTCAGCGATCTACATTAGCCGCATCCGGCATGAACAAAGCGCAGGAACAAGCGTCGCATCATGCCTCTTTGACCCACAGCTGCGGAAAACGTACTGGTGCAAAACGCA |
| D-lacIZYA-2 | CATGCATTTACGTTGACACCATCGAATGGCGCAAAACCTTTCGCGGTATGGCATGATAGCGCCCGGAAGAGAGTCAATTCAGGGTGGTGAATGTGAAACCAGTAACGTTATACGATGTCGCAGAGTATGCCGGTGTCTCTTATCAGACCGTTTCCCGCGTGGTGAACCAGGCCAGCCACGTTTCTGCGAAAACGCGGGAAAAAGTGGAAGCGGCGATGGCGGAGCTGACGTCCGATAACGATTGGCAATAACGTCTGGATCGGAAGTCATGTGGTTATTAATCCAGGCGTCACCATCGGGGATAATTCTGTTATTGGCGCGGGTAGTATCGTCACAAAAGACATTCCACCAAACGTCGTGGCGGCTGGCGTTCCTTGTCGGGTTATTCGCGAAATAAACGACCGGGATAAGCACTATTATTTCAAAGATTATAAAGTTGAATCGTCAGTTTAAATTATAAAAATTGCCTGATACGCTGCGCTTATCAGGC |

| | |
|---|---|
| metG630 | GCAGGCAATTTGCTCAATGGGCATCAACCTGTTCCGCGTGCTGATGAC<br>TTACCTGAAGCCGGTACTGCCGAAACTGACCGAGCGTGCAGAAGCATT<br>CCTCAATACGGAACTGACCTGGGATGGTATCCAGCAACCGCTGCTGGG<br>CCACAAAGTGAATCCGTTCAAGGCGCTGTATAACCGCATCGATATGAG<br>GCAGGTTGAAGCACTGGTGGAAGCCTCTAAAGAAGAAGTAAAAGCCGC<br>TGCCGCGCCGGTAACTGGCCCGCTGGCAGATGATCCGATTCAGGAAA<br>CCATCACCTTTGACGACTTCGCTAAAGTTGACCTGCGCGTGGCGCTGA<br>TTGAAAACGCAGAGTTTGTTGAAGGTTCTGACAAACTGCTGCGCCTGA<br>CGCTGGATCTCGGCGGTGAAAAACGCAATGTCTTCTCCGGTATTCGTT<br>CTGCTTACCCGGATCCGCAGGCACTGGCCGCCAGACGGTAATGGTGG<br>CTAACCTGGCACCACGTAAAATGCGCTTCGGTATCTCTGAAGGCATGG<br>TGATGGCTGCCGGTCCTGGCGGGAAAGATATTTTCCTGCTAAGCCCGG<br>ATGCCGGTGCTAAACCGGGTCATCAGGTGAAATAATCCCCCTTCAAGG<br>CGCTGCATCGACAGCGCCTTTTCTTTATAAATTCCTAAAGTTGTTTTCTT<br>GCGATTTTGTCTCTCTCTAACCCGCATAAATACTGGTAGCATCTGCATT<br>CAACTGGATAAAATTACAGGGATGCAGAATGAGACACTTTATCTATCAG<br>GACGAAAAATCACATAAATTCAGGGCAGTTGAGCAACAGGGAAACGAG<br>TTGCATATCAGTTGGGGAAAAGTTGGCACCAAAGGGCAAAGCCAGATA<br>AAAAGTTTTTCAGATGCTGCGGCAGCGGCAAAAGCGGAGCTTAAGCTG<br>ATTGCGGAGAAGGTGAAGAAGGGGTATGTGGAGCAAGCGAAGGATAA<br>TTCTTTGCAAGCTAGGGATGCAACAG |
| metG-ETIT | CGCAGTTGTACAAAACCTTCACTGATGCCGCTGAAGTGATTGGTGAAG<br>CGTGGGAAAGCCGTGAATTTGGTAAAGCCGTGCGCGAAATCATGGCG<br>CTGGCTGATCTGGCTAACCGCTATGTCGATGAACAGGCTCCGTGGGTG<br>GTGGCGAAACAGGAAGGCCGCGATGCCGACCTGCAGGCAATTTGCTC<br>AATGGGCATCAACCTGTTCCGCGTGCTGATGACTTACCTGAAGCCGGT<br>ACTGCCGAAACTGACCGAGCGTGCAGAAGCATTCCTCAATACGGAACT<br>GACCTGGGATGGTATCCAGCAACCGCTGCTGGGCCACAAAGTGAATC<br>CGTTCAAGGCGCTGTATAACCGCATCGATATGAGGCAGGTTGAAGCAC<br>TGGTGGAAGCCTCTAAAGAAGAAGTAAAAGCCGCTGCCGCGCCGGTA<br>ACTGGCCCGCTGGCAGATGATCCGATTCAGTTTGACGACTTCGCTAAA<br>GTTGACCTGCGCGTGGCGCTGATTGAAAACGCAGAGTTTGTTGAAGGT<br>TCTGACAAACTGCTGCGCCTGACGCTGGATCTCGGCGGTGAAAAACGC<br>AATGTCTTCTCCGGTATTCGTTCTGCTTACCCGGATCCGCAGGCACTG<br>ATTGGTCGTCACACCATTATGGTGGCTAACCTGGCACCACGTAAAATG<br>CGCTTCGGTATCTCTGAAGGCATGGTGATGGCTGCCGGTCCTGGCGG<br>GAAAGATATTTTCCTGCTAAGCCCGGATGCCGGTGCTAAACCGGGTCA<br>TCAGGTGAAATAATCCCCCTTCAAGGCGCTGCATCGACAGCGCCTTTT<br>CTTTATAAATTCCTAAAGTTGTTTTCTTGCGATTTTGTCTCTCTCTAACC<br>CGCATAAATACTGGTAGCATCTGCATTCAACTGGATAAAATTACAGGGA<br>TGCAGAATGAGACACTTTATCTATCAGGACGAAAAATCACATAAATTCA<br>GGGCAGTTGAGCAACAG |

**SI TABLE 3. Performance of RIPR gene editing of *lacZ*.**

| Mutation | Fraction of recombinants that lost Cam$^R$ plasmid cointegrate marker | Isolates having undergone allelic exchange (white/blue) | Isolates with predicted genotype confirmed by DNA sequencing |
|---|---|---|---|
| *lacZ571* (Glu-461-Am*) | 50/50 | 23/50 | 5/5 |
| *lacZ572* (Glu-461-Gly) | 50/50 | 30/50 | 5/5 |
| D*lacIZYA* (500-bp homology) | 50/50 | 19/50 | 5/5 |
| D*lacIZYA* (250-bp homology) | 50/50 | 21/50 | 5/5 |

## References


BLUMENTHAL, T. 1972. P1 transduction: formation of heterogenotes upon cotransduction of bacterial genes with a P2 prophage. *Virology,* 47**,** 76-93.

CUI, L., VIGOUROUX, A., ROUSSET, F., VARET, H., KHANNA, V. & BIKARD, D. 2018. A CRISPRi screen in E. coli reveals sequence-specific toxicity of dCas9. *Nat Commun,* 9**,** 1912.

CUPPLES, C. G. & MILLER, J. H. 1988. Effects of amino acid substitutions at the active site in Escherichia coli beta-galactosidase. *Genetics,* 120**,** 637-44.

CUPPLES, C. G. & MILLER, J. H. 1989. A set of lacZ mutations in Escherichia coli that allow rapid detection of each of the six base substitutions. *Proc Natl Acad Sci U S A,* 86**,** 5345-9.

FERRIERES, L., HEMERY, G., NHAM, T., GUEROUT, A. M., MAZEL, D., BELOIN, C. & GHIGO, J. M. 2010. Silent mischief: bacteriophage Mu insertions contaminate products of Escherichia coli random mutagenesis performed using suicidal transposon delivery plasmids mobilized by broad-host-range RP4 conjugative machinery. *J Bacteriol,* 192**,** 6418-27.

GREENE, R. C., HUNTER, J. S. & COCH, E. H. 1973. Properties of metK mutants of Escherichia coli K-12. *J Bacteriol,* 115**,** 57-67.

KIM, J., WEBB, A. M., KERSHNER, J. P., BLASKOWSKI, S. & COPLEY, S. D. 2014. A versatile and highly efficient method for scarless genome editing in Escherichia coli and Salmonella enterica. *BMC Biotechnol,* 14**,** 84.

LEVIN-REISMAN, I., RONIN, I., GEFEN, O., BRANISS, I., SHORESH, N. & BALABAN, N. Q. 2017. Antibiotic tolerance facilitates the evolution of resistance. *Science,* 355**,** 826-830.
LOVETT, S. T., HURLEY, R. L., SUTERA, V. A., JR., AUBUCHON, R. H. & LEBEDEVA, M. A. 2002. Crossing over between regions of limited homology in Escherichia coli. RecA-dependent and RecA-independent pathways. *Genetics,* 160**,** 851-9.
PHILLIPS, G. J., PARK, S. K. & HUBER, D. 2000. High copy number plasmids compatible with commonly used cloning vectors. *Biotechniques,* 28**,** 400-2, 404, 406 passim.

**SI Figure Legends**

**SI Figure 1. Results of genome editing of the *lac* operon by RIPR.** A. Single base pair substitutions in the wild type (WT) *lacZ* gene at the position encoding amino acid 461 leads to complete loss of β-galactosidase activity (*lacZ571*, E461Am) or partial activity (*lacZ572*, E461G) in presence of X-Gal. B. *E. coli* cells plated on X-Gal after counterselection by I-SceI expression leads to deletion of the *lac* operon in roughly half of the recombinants (white colonies) vs. retention of the wild type allele (blue colonies).

**SI Figure 2: Growth curves of different metG mutations that affect bacterial antibiotic persistence in *E.coli*.** All strains were grown in LB medium at 37 °C with shaking for 8 hours. Wild type, *metG87*, *metG83*, *metG630*, and *metG∆ETIT E. coli* strains were monitored by $OD_{600}$. *metG87* is a S264F point mutation near the catalytic site, *metG83* is a G263S/S264F double mutation near the catalytic site, *metG630* is a I626A frameshift mutation that results in a premature stop codon near the anticodon binding domain, *metG∆ETIT* is a deletion mutation from 569-572 near the anticodon binding domain. Error bars represent standard deviations from 3 biological replicates.

**SI Figure 3: Growth curves of aaRS knock down *E. coli* strains with varying concentrations of anhydrous tetracycline.** All strains were grown in LB medium at 37 °C with shaking for 20 hours, anhydrous tetracycline concentrations ranged from 0 – 4 mg/mL. A) wildtype, B) empty vector, C) *alaS* knock down, D) *metG* knock down *E. coli* strain. Empty vector contains the CRISPR-Cas9 plasmid with no guide RNA. Error bars represents standard deviations from 3 biological replicates.

**SI Figure 4: Growth curves and colony forming units of aaRS knock down *E. coli* strains after 3 h induction with varying concentrations of anhydrous tetracycline.** All strains were grown in LB medium at 37 °C with shaking until $OD_{600}$ nm ~ 0.1, anhydrous tetracycline concentrations ranging from 0 – 2.5 mg/mL was added, and strains continued to grow for 3 hours. A) Growth curves of wildtype, empty vector, alaRS knockdown, and *metG* knockdown *E. coli* strains after 3 h anhydrous tetracycline induction monitored at OD at 600 nm. B) Colony forming units of wildtype, empty vector, alaRS knockdown, and metG knockdown *E. coli* strains after 3 h anhydrous tetracycline induction. Empty vector contains no guide RNA. Error bars represents standard deviations from 3 biological replicates.

**SI Figure 5: There are no significant changes in polysome profiles of various metG mutation strains.** The various *metG* mutations were made on the *E. coli* chromosome so that

the corresponding MetRS mutation would be made *in vivo*. Cultures were grown in M9 medium supplemented with 50 mM methionine until an $OD_{600}$ of 0.7. Cells were lysed and loaded onto a sucrose gradient to measure 30S, 50S, 70S, and polysome fraction profiles. Error bars represents standard deviations from 3 biological replicates.

**SI Figure 6: MetRS mechanism with cognate amino acid, methionine, and non-cognate amino acids, homocysteine and S-NO-homocysteine.** The top reaction scheme shows the conical reaction of MetRS with methionine, the middle reaction mechanism shows the pre-transfer editing of homocysteine to release homocysteine thiolactone, the bottom reaction shows MetRS with S-NO-homocysteine.

**SI Figure 1. Results of genome editing of the *lac* operon by RIPR.**

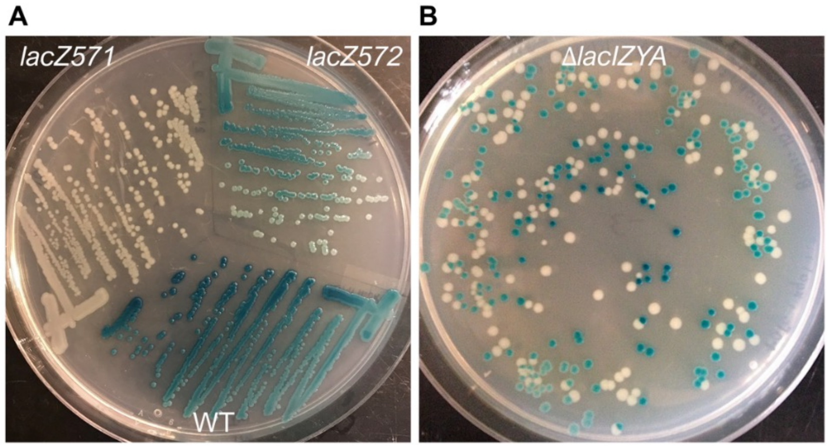

**SI Figure 2: Growth curves of different *metG* mutations that affect bacterial antibiotic persistence in *E.coli*.**

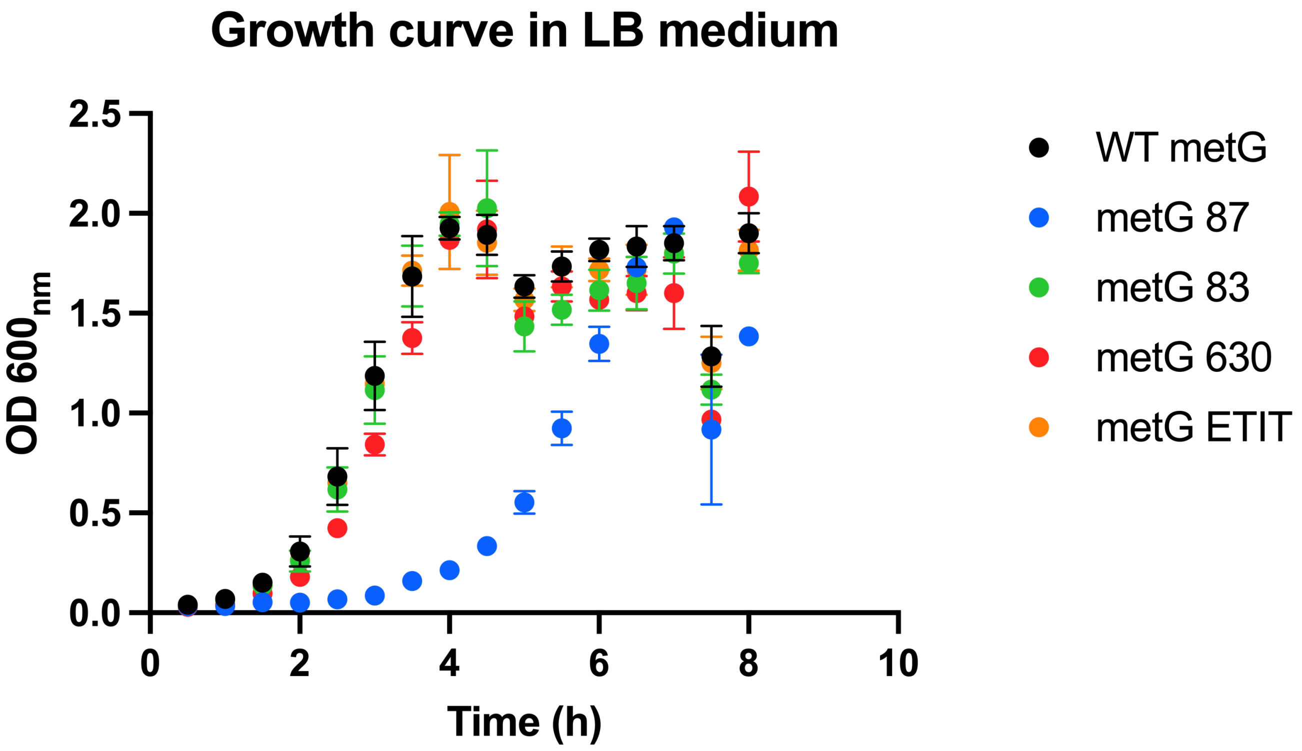

**SI Figure 3: Growth curves of aaRS knock down *E. coli* strains with varying concentrations of anhydrous tetracycline.**

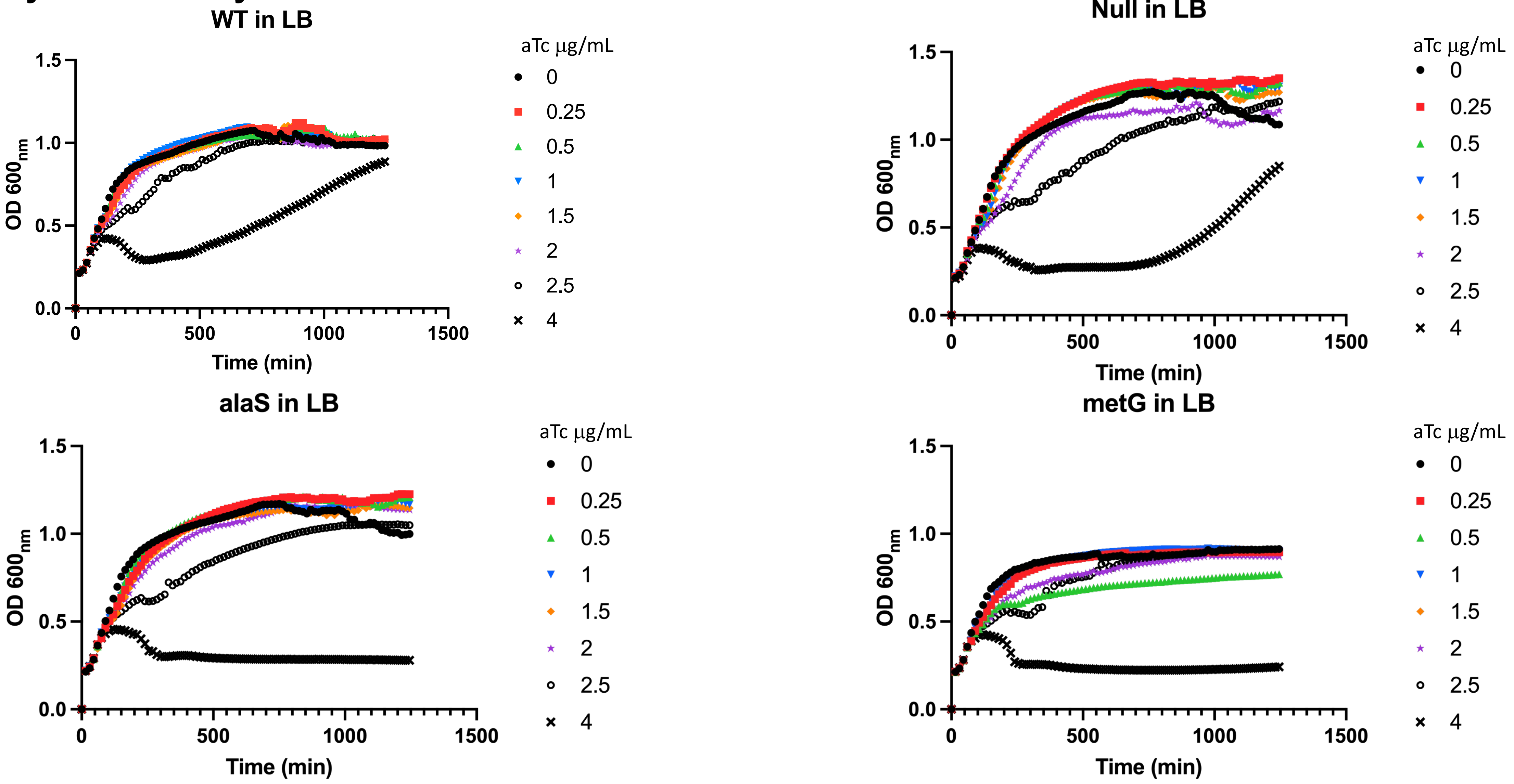

**SI Figure 4: Growth curves and colony forming units of aaRS knock down *E. coli* strains after 3 h induction with varying concentrations of anhydrous tetracycline.**

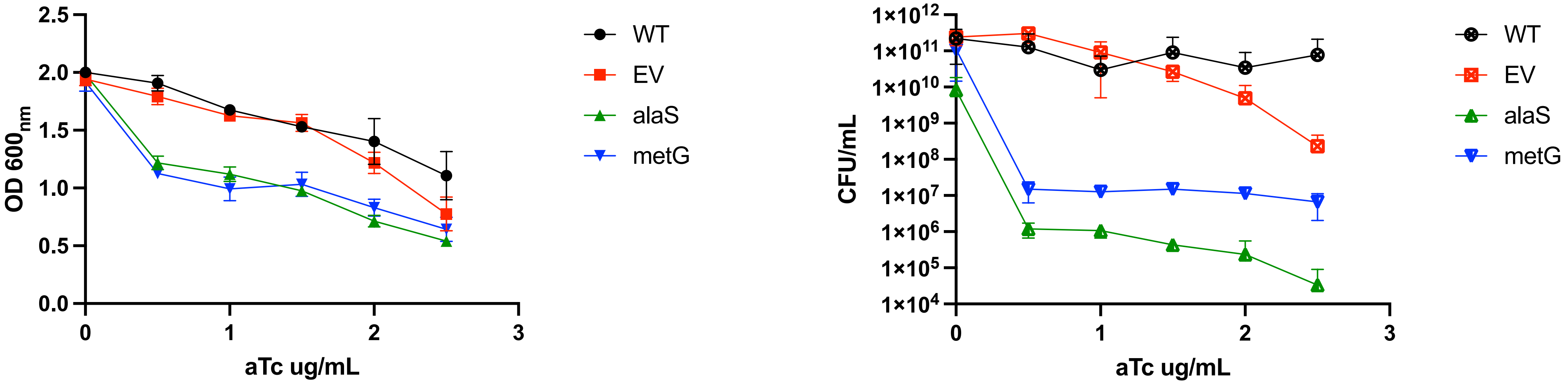

**SI Figure 5: There is no significant changes in polysome profiles of various *metG* mutation strains.**

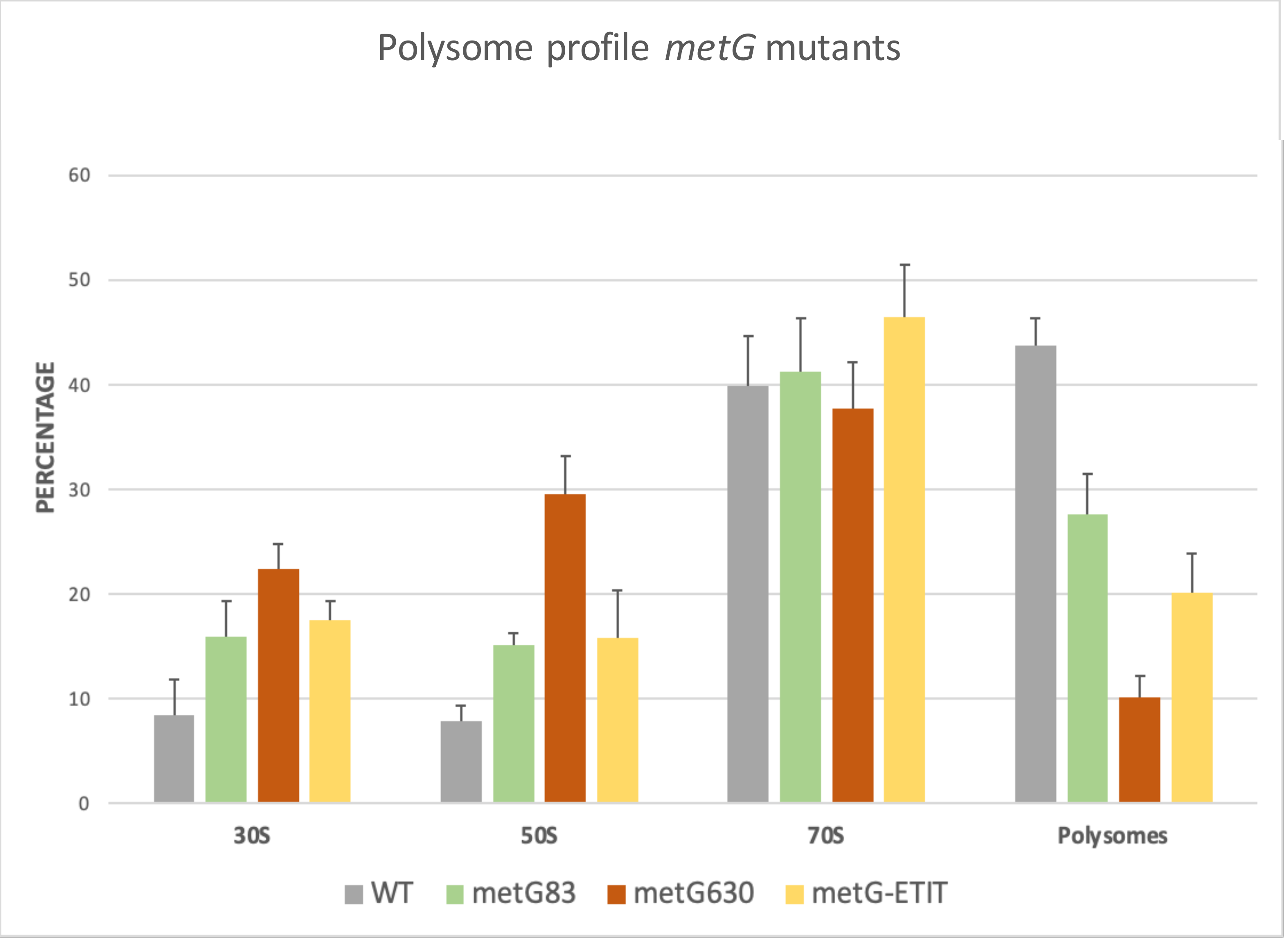

**SI Figure 6: MetRS mechanism with cognate amino acid, methionine, and non-cognate amino acids, homocysteine and S-NO-homocysteine.**

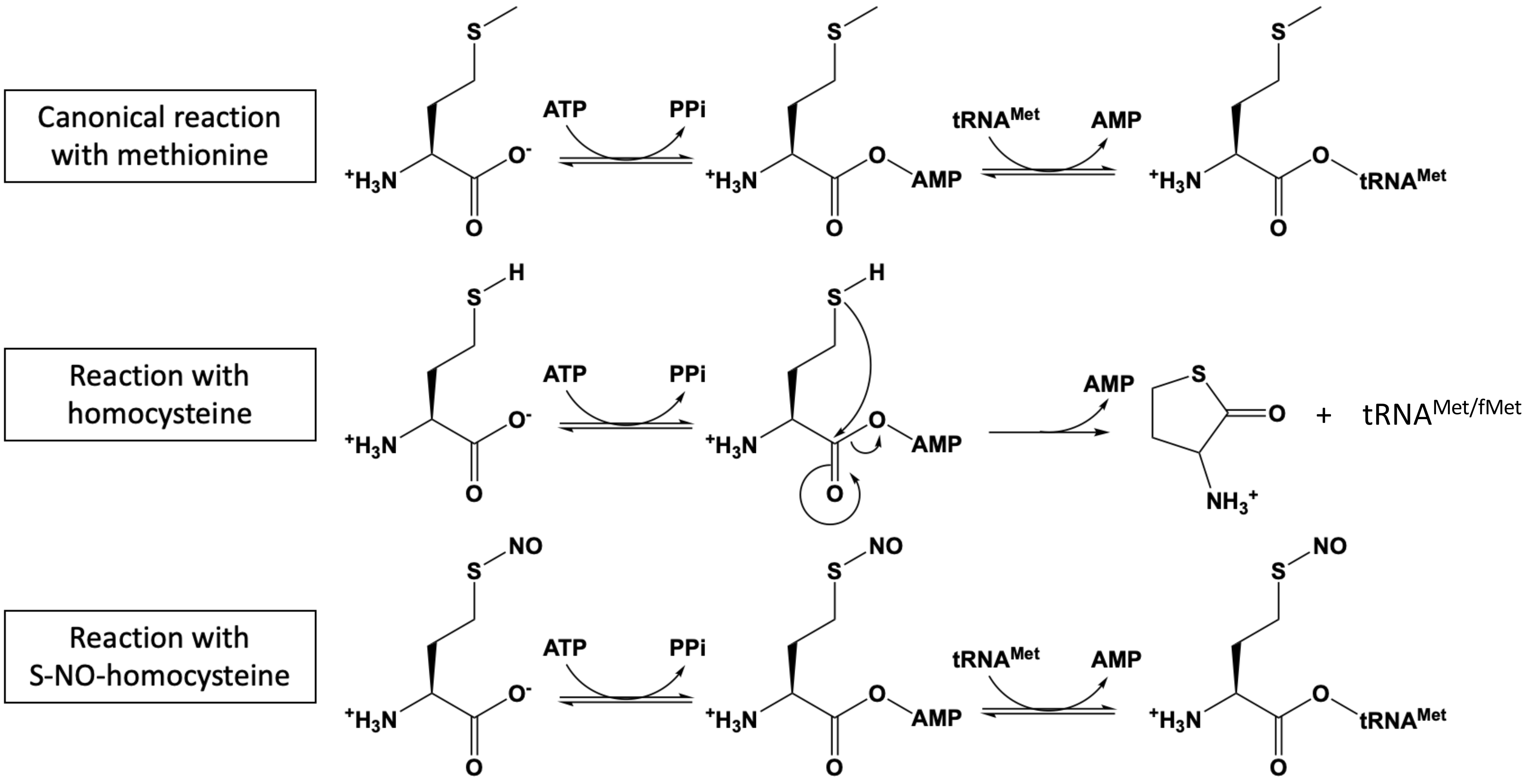

Supplement: Supplementary file 1 [file Data_Sheet_1.PDF]
